# Pathogen spectrum and epidemiology of respiratory tract infections in Quzhou, Eastern China, from November 2023 to July 2024: A post‑COVID‑19 surveillance study

**DOI:** 10.1371/journal.pone.0346441

**Published:** 2026-06-08

**Authors:** Rui-jun Yang, Min Wang, Lei Lyu, Jia-ling You, Shi-teng Huang, Bing-dong Zhan

**Affiliations:** Quzhou Center for Disease Control and Prevention, Quzhou, Zhejiang, China; Children's National Hospital, George Washington University, UNITED STATES OF AMERICA

## Abstract

**Background:**

After the relaxation of COVID‑19 containment measures, we investigated the pathogen spectrum and epidemiological characteristics of acute respiratory infections (ARIs) in Quzhou City from 2023 to 2024.

**Objective:**

This study aimed to investigate the pathogen spectrum and epidemiological characteristics of acute respiratory infections (ARIs) in Quzhou City from 2023 to 2024, providing a scientific basis for local prevention and control strategies.

**Methods:**

A total of 2,800 respiratory specimens were collected from November 2023 to July 2024, comprising 1,960 influenza-like illness (ILI) cases from outpatient/emergency departments and 840 severe acute respiratory infection (SARI) cases from inpatient departments. All samples were tested for 13 common respiratory pathogens using multiplex fluorescence quantitative PCR. Etiological and epidemiological analyses were performed based on detection results and case information.

**Results:**

The overall ARI positivity rate was 59.28% (1,660/2,800), with a male-to-female ratio of 1.07:1 (1,447/1,353). The three most prevalent pathogens were influenza virus (Flu, 23.21%, 650/2,800), Streptococcus pneumoniae (SP, 13.14%, 368/2,800), and adenovirus (ADV, 8.39%, 235/2,800). Single pathogen infections accounted for 73.55% (1,221/1,660) of positive cases, while co-infections with two or more pathogens accounted for 26.45% (439/1,660), yielding an overall co-infection rate of 15.68% (439/2,800). No significant gender difference was observed in detection rates. However, significant differences were found across case types, temporal periods, age groups, and geographic regions (P < 0.01). Children aged ≤5 years exhibited the highest positivity rate (78.00%, 378/525), while adults aged ≥65 years showed the lowest (34.53%, 144/417). Among surveillance regions, Kaihua County had the highest positivity rate (72.47%), and Changshan County the lowest (40.55%).

**Conclusions:**

Multiple respiratory pathogens and co-infections are prevalent in Quzhou City, with distinct age-specific and seasonal patterns. These findings underscore the need for continuous multi-pathogen surveillance and integrated prevention strategies for influenza and other respiratory infectious diseases in the post-pandemic era.

## 1 Introduction

Acute respiratory infections (ARIs) represent a significant global public health burden, ranking as the fourth leading cause of mortality worldwide [1]. The pathogen spectrum of respiratory tract infections is complex and dynamic, encompassing a wide array of etiological agents including viruses, bacteria, and atypical pathogens such as Mycoplasma pneumoniae and Chlamydia pneumoniae. These pathogens can circulate either independently or concomitantly, potentially leading to local outbreaks or even global pandemics [2].

In recent decades, the landscape of respiratory pathogens has been continually reshaped by the emergence of novel agents, including human metapneumovirus (HMPV) [3], human bocavirus (HBoV), and most notably, the unprecedented COVID-19 pandemic caused by SARS-CoV-2 [4]. The implementation of stringent non-pharmaceutical interventions during the pandemic dramatically altered the circulation patterns of common respiratory pathogens globally, leading to historically low detection rates of influenza, respiratory syncytial virus (RSV), and other endemic viruses [4]. This disruption of established epidemiological patterns has raised concerns about potential “immunity debt”—particularly among young children who experienced reduced exposure to common respiratory pathogens during the pandemic [5].

Following the relaxation of COVID-19 containment measures in China since early 2023, the country has witnessed a resurgence and alteration in the transmission dynamics of various respiratory pathogens. However, limited data are available regarding the post-pandemic epidemiological characteristics of respiratory pathogens at the regional level, particularly in medium-sized cities in Eastern China [6–8]. Understanding these patterns is crucial for optimizing diagnostic strategies, guiding empirical treatment, and implementing targeted prevention measures.

Quzhou City, located in western Zhejiang Province, has established a multi-pathogen surveillance system for acute respiratory infections. This study analyzed surveillance data from 2,800 ARI cases collected between November 2023 and July 2024, aiming to characterize the pathogen spectrum, epidemiological features, and age-specific distribution of respiratory pathogens in the post-COVID-19 era. Our findings will provide a scientific basis for the rapid and accurate diagnosis, prevention, and control of respiratory infectious diseases in the region.

## 2 Materials and methods

### 2.1 Ethics statement

This study was conducted in accordance with the Declaration of Helsinki. The study protocol was reviewed and approved by the Ethics Committee of Quzhou Center for Disease Control and Prevention (Approval No.: Lun Shen 2024 Yan Di 018/ IRB-2024-R-018; date of approval: 24 September 2024).

The study involved a retrospective analysis of residual clinical specimens and anonymized medical records collected from patients presenting with acute respiratory infections between November 2023 and July 2024. All specimens and data were originally obtained for routine clinical diagnosis and surveillance purposes. Before inclusion in this study, all patient identifiers were removed, and the data were anonymized.

Because of the retrospective design and the use of fully anonymized data, the ethics committee granted a waiver of written informed consent. The ethics committee confirmed that no additional patient contact or prospective intervention was required. The study posed no more than minimal risk to patients, and the waiver did not adversely affect the rights or welfare of the subjects.

Regarding the timing of ethical approval: Although the data were collected prior to the approval date (24 September 2024), the study was composed entirely of retrospective, de-identified data. The ethics committee reviewed and approved the use of these pre-existing data retrospectively, which is consistent with standard practice for non-interventional, retrospective studies. The approval was obtained before any data analysis or manuscript preparation took place (data were accessed for research purposes on 15 January 2025).

### 2.2. Materials

#### 2.2.1 Study subjects and data collection.

A total of 1,960 influenza-like illness (ILI) cases from outpatient and emergency departments, and 840 severe acute respiratory infections (SARI) cases from inpatient departments were enrolled from county/district people’s hospitals across Quzhou City between November 2023 and July 2024. Demographic and clinical information of the enrolled cases was recorded in detail. The inclusion criteria [9] were: (1) ILI (outpatient/emergency): Fever (axillary temperature ≥ 38°C) with onset within the last 10 days, and at least one of the following: cough or sore throat.(2) SARI (inpatient): Fever (≥38°C) with onset within the last 10 days, cough or sore throat, and clinical evidence of lower respiratory tract involvement (e.g., shortness of breath, tachypnea, abnormal breath sounds on auscultation) requiring hospital admission, as documented by the attending physician. These criteria follow the WHO SARI case definition and the Zhejiang Provincial Multi‑pathogen Surveillance Protocol.The case enrollment principles complied with the “Notice on the issuance of the Zhejiang Province Acute Respiratory Infection Multi-pathogen Surveillance Protocol (Trial)” issued by the Comprehensive Office of the Zhejiang Provincial Disease Control and Prevention Bureau. Enrollment and sample size justification: All consecutive ARI patients meeting the case definitions at the participating county‑level people’s hospitals during the study period (November 2023 – July 2024) were included. No additional sampling or selection was applied. The ratio of ILI to SARI (1,960:840) reflects the natural distribution of outpatient/emergency versus hospitalized ARI cases in our surveillance network. A post‑hoc power analysis showed that with 2,800 cases, the study had 84% power to detect a 10% absolute difference in positivity rates between subgroups (α = 0.05, two‑sided).This study was designed as a pathogen-based surveillance investigation and did not systematically collect clinical outcome data, including hospitalization duration, oxygen requirement, intensive care unit (ICU) admission, or mortality.

#### 2.2.2 Specimen collection.

(1) For ILI cases: Throat swabs, nasal swabs, or nasopharyngeal swabs were collected within 3 days of illness onset. After collection, specimens were placed into collection tubes containing 3–4 mL of viral transport medium.
(2) For SARI cases: Respiratory specimens, including throat swabs, nasal swabs, nasopharyngeal swabs, bronchoalveolar lavage fluid, and pleural puncture fluid, were collected from patients admitted to the hospital due to ARI, within 3 days of illness onset. After collection, specimens were placed into collection tubes containing 3–4 mL of viral transport medium.

#### 2.2.3 Instruments and reagents.

ABI real-time fluorescent PCR instruments and a ZY Biotechnology nucleic acid extraction system were used. PCR detection was performed using a 13-pathogen nucleic acid detection kit (Fluorescence PCR method) manufactured by Jiangsu Bioperfectus Technologies Co., Ltd.It should be noted that PCR is one type of nucleic acid amplification testing (NAAT), specifically a thermal cycling-based method. Isothermal NAATs, such as loop-mediated isothermal amplification (LAMP) and recombinase polymerase amplification (RPA), are also widely used for rapid molecular detection of some respiratory pathogens [10].

#### 2.2.4 Quality control and Ct value threshold.

For each PCR run, a positive control (a mixture of synthetic nucleic acid fragments of the 13 targets at known concentration) and a negative control (RNase‑free water) were included. A sample was considered positive if the cycle threshold (Ct) value was < 35. Samples with Ct values between 35 and 38 were re‑tested; if the repeat Ct remained <38, they were reported as borderline positive. No template control and no‑reverse‑transcriptase control were used for RNA pathogens. Inter‑run variability was assessed by calculating the coefficient of variation of Ct values of the positive control across runs (CV < 5% for all targets).

#### 2.2.5 Specimen types and sensitivity analysis.

Among SARI cases, the majority (83.6%, 702/840) were throat swabs; a minority were bronchoalveolar lavage fluid (BALF, n = 130) and pleural fluid (n = 8). Due to the small number of non-throat swab specimens (n = 138, accounting for 16.4%), formal statistical comparison of detection rates between different specimen types was not performed. The potential impact of specimen type heterogeneity on diagnostic sensitivity is acknowledged as a limitation of this study (see Discussion)..

### 2.3 Methods

Specimens were transported to the virology laboratory at 4°C immediately after collection. Real-time RT-PCR was performed promptly to detect nucleic acids of influenza virus (Flu), Mycoplasma pneumoniae (MP), Streptococcus pneumoniae (SP), SARS-CoV-2, rhinovirus (RV), human metapneumovirus (HMPV), human adenovirus (AdV), respiratory syncytial virus (RSV), human parainfluenza virus (HPIV), human coronavirus (HCoV), human bocavirus (HBoV), Chlamydia pneumoniae (CP), and enterovirus (EV).

### 2.4 Statistical analysis

Data were collated using Excel and analyzed using SPSS 21.0 software. Comparisons of rates among multiple groups were performed using the chi-square test (χ² test). A trend test was used to analyze the association between age and the detection rate of co-infections. A P-value <0.05 was considered statistically significant.To identify independent predictors of overall pathogen positivity, we performed multivariable binary logistic regression analysis. The dependent variable was positivity (positive vs. negative for any of the 13 pathogens). Independent variables included age group (≤5, 6–14, 15–64, and ≥65 years, with ≥65 as reference), sex (male vs. female), case type (ILI vs. SARI), month (as a continuous variable from 1 [November 2023] to 9 [July 2024]), and surveillance region (six counties/districts, with Changshan as reference). Adjusted odds ratios (ORs) with 95% confidence intervals (CIs) were calculated. A two-sided P < 0.05 was considered statistically significant. The model fit was assessed using the Hosmer-Lemeshow test.Given the exploratory nature of this study, no correction for multiple comparisons was applied; however, findings with borderline P values should be interpreted cautiously.

## 3 Results

### 3.1 Baseline characteristics

From November 2023 to July 2024, a total of 2,800 acute respiratory infection (ARI) surveillance cases were enrolled, including 1,960 ILI cases (70.0%) and 840 SARI cases (30.0%). Among these, there were 1,447 males(51.7%) and 1,353 females(48.3%), with a male-to-female ratio of 1.07:1. The age distribution was as follows: ≤ 5 years (n = 525, 18.8%), 6–14 years (n = 713, 25.5%), 15–64 years (n = 1,145, 40.9%), and ≥65 years (n = 417, 14.9%).

### 3.2 Etiological characteristics

#### 3.2.1 Overall pathogen detection.

Nucleic acid testing for 13 common respiratory pathogens was performed on all 2,800 enrolled cases. A total of 1,660 cases (59.28%, 1,660/2,800) tested positive for ARI pathogens. The detection rate in ILI cases (64.39%) was significantly higher than that in SARI cases (47.38%) (P < 0.01). No statistically significant difference in pathogen detection was observed between sexes (P = 0.545). However, significant differences in detection rates were found among different age groups (P < 0.01). The highest positivity rate was observed in the 0–5 years age group (78.00%, 378/525), while the lowest was in the > 65 years age group (34.53%, 144/417). A significant linear trend was observed, indicating that the positivity rate decreased with increasing age (χ² = 172.511, P < 0.01). Furthermore, pathogen detection rates varied significantly across different months (P < 0.01). The baseline characteristics of the monitored cases are presented in Table 1.

**Table 1:** Baseline characteristics of acute respiratory infection surveillance cases in Quzhou City.

Characteristic	Total cases	No. positive	Positive rate (%)	χ²	*P*-value
Case type				70.457	<0.01
ILI	1960	1262	64.39		
SARI	840	398	47.38		
Sex				0.366	0.545
Male	1447	850	58.74		
Female	1353	810	59.87		
Age group(years)				200.315	<0.01
≦5	525	378	78.00		
6 ~ 14	713	514	72.09		
15 ~ 64	1145	624	54.50		
≧65	417	144	34.53		
Month				93.359	<0.01
11	275	198	72.00		
12	365	253	69.32		
1	463	280	60.48		
2	377	246	65.26		
3	381	225	59.06		
4	437	204	46.68		
5	382	181	47.38		
6	90	51	56.67		
7	30	22	73.33		
Total	2800	1660	59.28		

#### 3.2.2 Detection rates of specific pathogens.

Among the 2,800 cases, the detection rates of SARS-CoV-2, influenza virus (Flu), respiratory syncytial virus (RSV), human adenovirus (AdV), human metapneumovirus (HMPV), rhinovirus (RV), human parainfluenza virus (HPIV), human coronavirus (HCoV), human bocavirus (HBoV), enterovirus (EV), Mycoplasma pneumoniae (MP), Chlamydia pneumoniae (CP), and Streptococcus pneumoniae (SP) are shown in Table 2. Influenza virus exhibited the highest detection rate (23.21%), followed by Streptococcus pneumoniae (13.14%) and human adenovirus (8.39%), ranking second and third, respectively.

**Table 2:** Detection of pathogens in acute respiratory infection surveillance cases in Quzhou City.

Pathogen	No.positive	Detection rate(%)
SARS-CoV-2	153	5.46
influenza virus (Flu)	650	23.21
Respiratory syncytial virus (RSV)	107	3.82
Human adenovirus (AdV)	235	8.39
Human metapneumovirus (HMPV)	81	3.00
Rhinovirus (RV)	225	8.04
Human parainfluenza virus (HPIV)	55	1.96
Human coronavirus (HCoV)	51	1.82
Human bocavirus (HBoV)	10	0.36
Enterovirus (EV)	52	1.86
Mycoplasma pneumoniae (MP)	174	6.21
Chlamydia pneumoniae (CP)	22	0.78
Streptococcus pneumoniae (SP)	368	13.14

(Note: Among the 1,660 ARI-positive patients, 1,221 (73.55%, 1,221/1,660) tested positive for a single pathogen, and 439 (26.45%, 439/1,660) tested positive for two or more pathogens, yielding a co-infection detection rate of 15.68% (439/2,800). Analysis of the 439 co-infected cases revealed distinct patterns by number of pathogens. Among dual infections, the most frequent combinations were rhinovirus + Streptococcus pneumoniae (RV + SP), adenovirus + SP (AdV + SP), and respiratory syncytial virus + SP (RSV + SP). For triple infections, the predominant combinations were SARS-CoV-2 + RV + SP and SP + RV + AdV. Quadruple and higher-order co-infections were also observed, including RSV + RV + Mycoplasma pneumoniae + SP, and RSV + influenza virus + MP + SP + human coronavirus (HCoV).Streptococcus pneumoniae (SP) is a common colonizer of the nasopharynx; therefore its detection by PCR does not necessarily indicate invasive or infectious disease. The reported rate (13.14%) may overestimate the true burden of SP‑attributable acute respiratory infection.)

### 3.3. Epidemiological characteristics

#### 3.3.1 Age distribution.

The difference in pathogen detection rates among different age groups was statistically significant (χ² = 200.315, P < 0.01). The highest positivity rate was observed in children aged 5 years and below (78.00%, 378/525), while the lowest was in cases aged over 65 years (34.53%, 144/417). Among children aged 5 years and below, Streptococcus pneumoniae (SP) showed the highest detection rate (20.00%), followed by influenza virus (FLU) (15.24%) and adenovirus (ADV) (13.90%). In contrast, influenza virus (FLU) was the most frequently detected pathogen in the adolescent group (6–14 years), adult group (15–64 years), and elderly group (>65 years), with detection rates of 27.21%, 28.65%, and 11.51%, respectively. The differences in pathogen detection rates across all age groups were statistically significant (P < 0.05). Detailed pathogen detection results for different age groups are presented in Table 3. The co-infection rate also decreased significantly with age (Cochran-Armitage trend test: χ² = 41.2, P < 0.001), from 22.5% in children ≤5 years to 8.3% in elderly ≥65 years.

**Table 3:** Pathogen detection in ARI cases by age group [n (%)].

Pathogen	total (n = 2800)	≦5 yrs (n = 525)	6 ~ 14 yrs (n = 713)	15 ~ 64 yrs (n = 1145)	≧65 yrs (n = 417)	χ²	*P*-value
Overall positive	1660(59.28)	378(78.00)	514(72.09)	624(54.50)	144(34.53)	200.315	<0.01
Flu	650(23.21)	80(15.24)	194(27.21)	328(28.65)	48(11.51)	76.117	<0.01
Influenza A	360(12.86)	35(6.67)	114(15.99)	180(15.72)	31(7.43)	43.523	<0.01
Influenza B	290(10.36)	45(8.57)	80(11.22)	148(12.92)	17(4.08)	28.227	<0.01
MP	174(6.21)	41(7.81)	100(14.02)	29(2.53)	4(0.96)	123.319	<0.01
SP	368(13.14)	105(20.00)	130(18.23)	112(9.78)	21(5.04)	73.146	<0.01
SARS-CoV-2	153(5.46)	11(2.10)	29(4.07)	83(7.25)	30(7.19)	23.705	<0.01
RV	225(8.04)	65(12.38)	72(10.10)	68(5.94)	20(4.80)	30.252	<0.01
HMPV	81(3.00)	25(4.76)	24(3.37)	22(1.92)	10(2.40)	11.307	0.01
ADV	235(8.39)	73(13.90)	111(15.57)	40(3.49)	11(2.64)	122.200	<0.01
RSV	107(3.82)	65(12.38)	25(4.76)	12(2.28)	5(0.95)	136.611	<0.01
HPIV	55(1.96)	24(4.57)	12(1.68)	12(1.05)	7(1.68)	23.992	<0.01
HCoV	51(1.82)	17(3.24)	13(1.82)	15(1.31)	6(1.44)	7.908	<0.05
HBoV	10(0.36)	7(1.33)	1(0.14)	2(0.17)	0(0.00)	11.456	<0.01
CP	22(0.78)	5(0.95)	12(1.68)	4(0.35)	1(0.24)	10.641	<0.05
EV	52(1.86)	29(5.52)	18(2.52)	3(0.26)	2(0.48)	60.794	<0.01

(Note: Flu: influenza virus; MP: Mycoplasma pneumoniae; SP: Streptococcus pneumoniae; SARS-CoV-2: severe acute respiratory syndrome coronavirus 2; RV: rhinovirus; HMPV: human metapneumovirus; AdV: human adenovirus; RSV: respiratory syncytial virus; HPIV: human parainfluenza virus; HCoV: human coronavirus; HBoV: human bocavirus; CP: Chlamydia pneumoniae; EV: enterovirus.)

**Fig 1:**
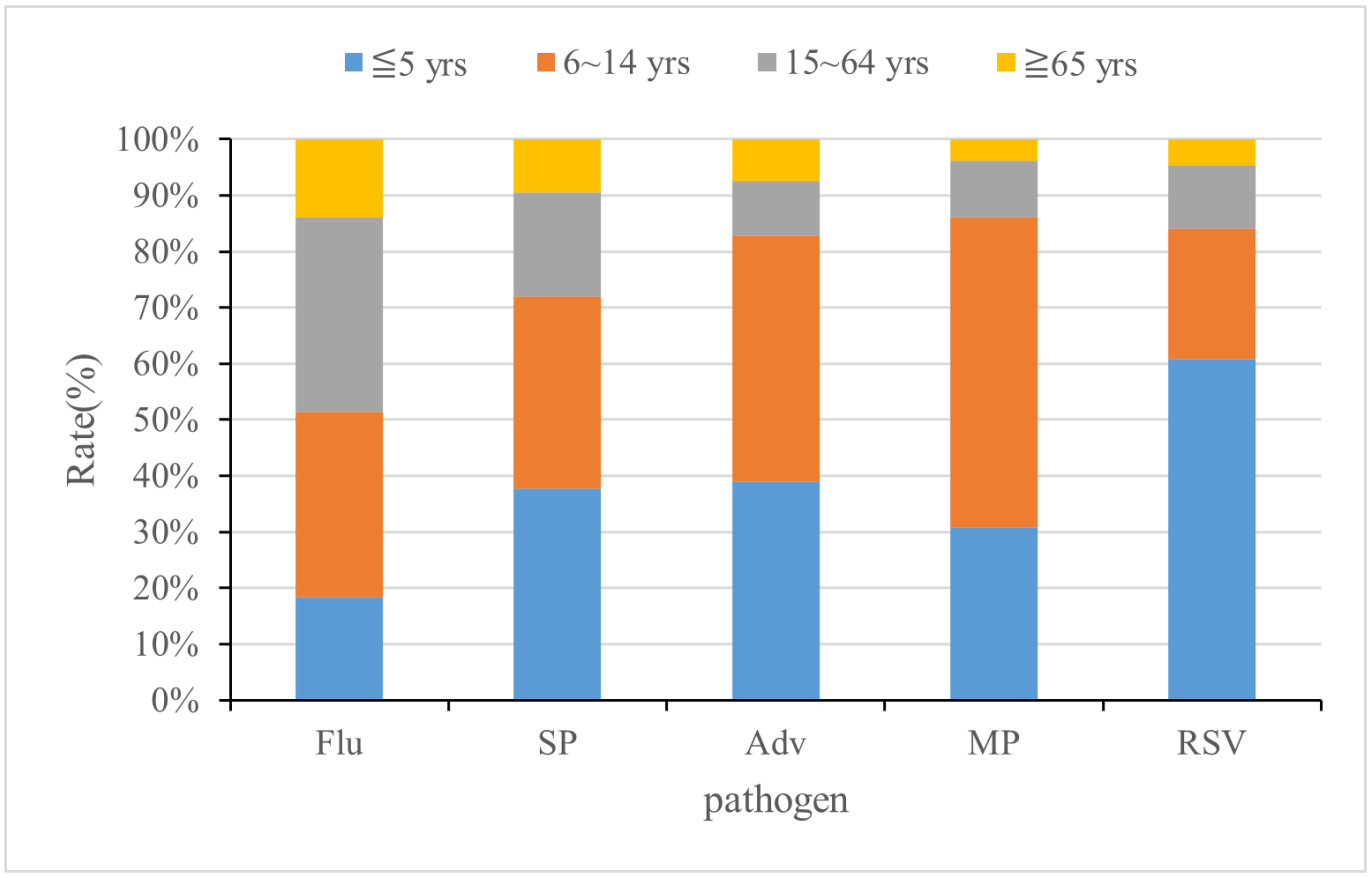
Positivity rates of common respiratory pathogens across different age groups in Quzhou City (November 2023 – July 2024). Abbreviations: Flu, influenza virus; SP, Streptococcus pneumoniae; AdV, human adenovirus; MP, Mycoplasma pneumoniae; RSV, respiratory syncytial virus.

The positivity rates of common pathogens in different age groups are shown in Fig 1.

#### 3.3.2 Time distribution.

From a temporal perspective, the monthly positive rate of pathogen detection throughout the surveillance period from November 2023 showed an overall trend of initially decreasing, then increasing, followed by another decrease, with inflection points occurring in February 2024 and April 2024. Specifically, the dominant pathogen, influenza A virus, exhibited peak detection rates in November (45.45%) and December (43.29%), subsequently returning to relatively stable levels. Influenza B virus showed peak detection rates in January (27.86%) and February (29.71%). Streptococcus pneumoniae (SP) was detected at relatively high levels throughout the entire study period. Adenovirus (AdV) showed a peak detection period from March to May. Additionally, SARS-CoV-2 exhibited a peak between February and March. In April, all 13 pathogens were detected. The temporal distribution characteristics of various pathogens detected in the 2,800 acute respiratory infection surveillance cases are presented in Table 4.

**Table 4:** Temporal distribution of pathogen detection in ARI cases in Quzhou City.

Month	Total cases	No.positive	Positive rate%	Flu (%)	MP (%)	SP (%)	SARS-COV-2 (%)	RV (%)	HMPV (%)	ADV (%)	RSV (%)	HPIV (%)	HCOV (%)	HBOV (%)	CP (%)	EV (%)
Nov	275	198	72	127 (46.18)	33 (12.00)	54 (19.64)	2 (0.73)	20 (7.27)	18 (6.55)	6 (2.18)	3 (1.09)	7 (2.55)	7 (2.55)	3 (1.09)	0 (0.00)	0 (0.00)
Dec	365	253	69.32	171 (46.85)	39 (10.68)	50 (13.70)	0 (0.00)	12 (3.29)	20 (5.48)	7 (1.92)	1 (0.27)	3 (0.82)	15 (4.11)	2 (0.55)	0 (0.00)	1 (0.27)
Jan	463	280	60.48	179 (38.66)	30 (6.48)	56 (12.09)	19 (4.10)	15 (3.24)	21 (4.54)	15 (3.24)	12 (2.59)	8 (1.73)	9 (1.94)	0 (0.00)	2 (0.43)	2 (0.43)
Feb	377	246	65.26	117 (31.03)	17 (4.51)	41 (10.88)	43 (11.14)	29 (7.69)	9 (2.39)	18 (4.77)	18 (4.77)	7 (1.86)	7 (1.86)	1 (0.27)	0 (0.00)	1 (0.27)
Mar	381	225	59.06	41 (10.65)	16 (4.20)	34 (8.92)	50 (13.12)	33 (8.66)	7 (1.84)	35 (9.19)	48 (12.60)	6 (1.57)	3 (0.78)	0 (0.00)	5 (1.31)	2 (0.53)
Apr	437	204	46.68	7 (1.60)	19 (4.35)	51 (11.67)	14 (3.20)	47 (10.76)	6 (1.37)	82 (18.76)	18 (4.12)	11 (2.52)	1 (0.23)	2 (0.46)	6 (1.37)	6 (1.37)
May	382	181	47.38	7 (1.83)	14 (3.66)	61 (16.00)	16 (4.19)	53 (13.87)	0 (0.00)	56 (14.66)	7 (1.83)	7 (1.83)	7 (1.83)	2 (0.52)	2 (0.52)	24 (6.28)
Jun	90	51	56.67	7 (7.78)	4 (4.44)	14 (15.56)	2 (2.22)	12 (13.33)	0 (0.00)	15 (16.67)	0 (0.00)	4 (4.44)	1 (1.11)	0 (0.00)	6 (6.67)	10 (11.11)
Jul	30	22	73.33	7 (23.33)	2 (6.67)	7 (23.33)	7 (23.33)	4 (13.33)	0 (0.00)	1 (3.33)	0 (0.00)	2 (6.67)	1 (3.33)	0 (0.00)	1 (3.33)	6 (20.00)

(Note: Abbreviations are as in Table 3).

**Fig 2:**
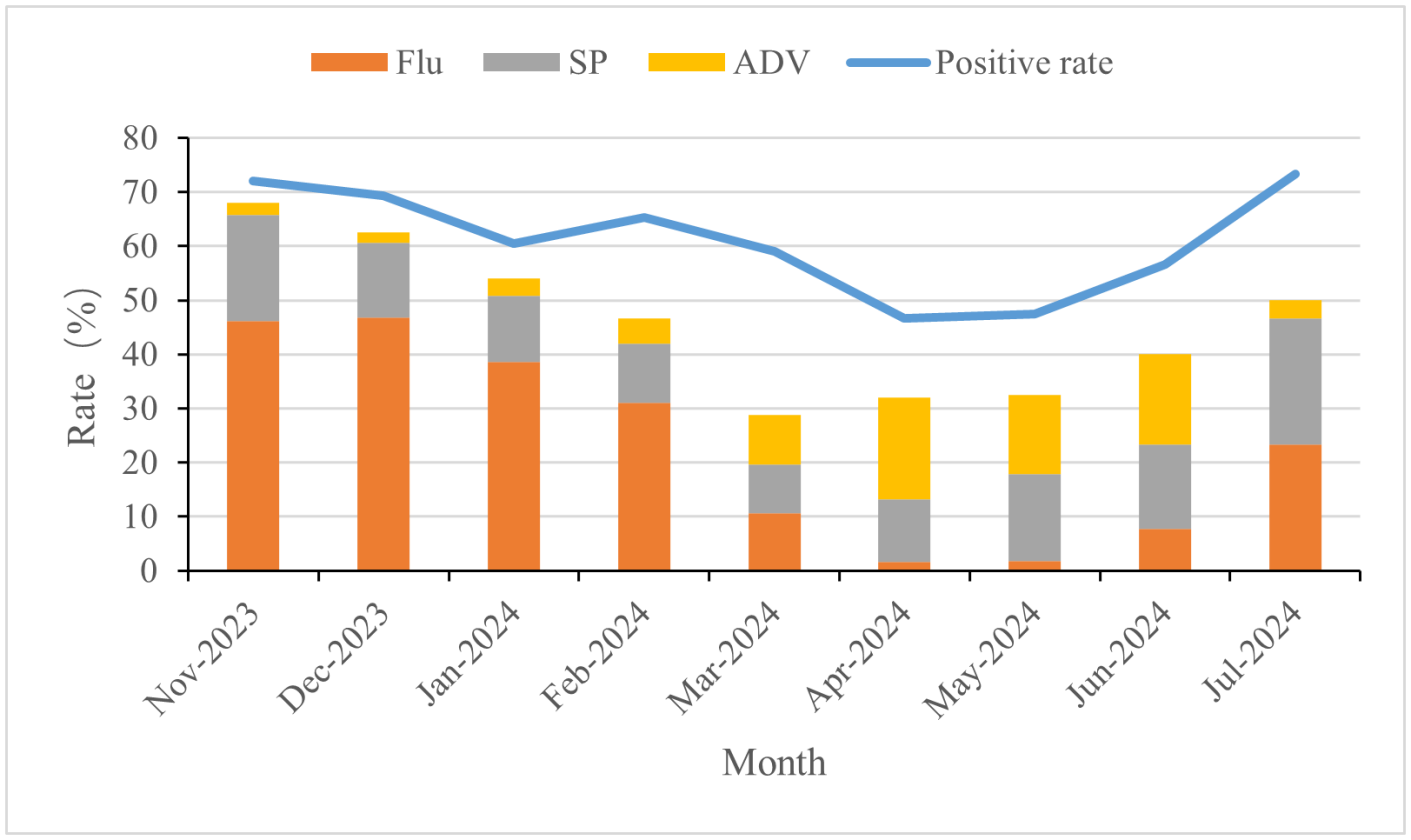
Monthly positivity rates of major respiratory pathogens from November 2023 to July 2024 in Quzhou City. The x-axis represents months, and the y-axis represents positivity rate (%). Different pathogens are distinguished by colored lines as shown in the legend.

The trend of monthly positivity rates of major pathogens from November 2023 to July 2024 is shown in Fig 2.

#### 3.3.3 Geographic distribution.

Analysis of pathogen detection across different counties and districts revealed the following: In Kecheng District, 360 ARI cases tested positive (66.67%, 360/540), with the top three pathogens being Flu (25.74%, 139/540), SP (22.78%, 123/540), and RV (11.30%, 61/540). In Qujiang District, 261 ARI cases tested positive (57.49%, 261/454), with the top three pathogens being Flu (22.90%, 104/454), SP (12.56%, 57/454), and RV (10.79%, 49/454). In Longyou County, 361 ARI cases tested positive (67.10%, 361/538), with the top three pathogens being Flu (24.54%, 132/538), SP (13.57%, 73/538), and RV (10.78%, 58/538). In Jiangshan City, 171 ARI cases tested positive (45.60%, 171/375), with the top three pathogens being Flu (22.40%, 84/375), AdV (5.07%, 19/375), and SP (3.73%, 14/375). In Changshan County, 178 ARI cases tested positive (40.55%, 178/439), with the top three pathogens being Flu (18.91%, 83/439), SP (7.74%, 34/439), and SARS-CoV-2 (6.15%, 27/439). In Kaihua County, 329 ARI cases tested positive (72.47%, 329/454), with the top three pathogens being Flu (23.79%, 108/454), AdV (20.48%, 93/454), and SP (14.76%, 67/454).Notably, Kaihua County exhibited the highest positivity rate (72.47%), while Changshan County had the lowest (40.55%). Furthermore, the difference in respiratory multi-pathogen positivity rates among the surveillance regions was statistically significant (χ² = 152.049, P < 0.01).

### 3.4 Comparison of pathogen detection between ILI and SARI cases

Overall, the difference in pathogen detection rates between ILI and SARI cases was statistically significant (χ² = 70.457, P < 0.01). Specifically, statistically significant differences (P < 0.05) between ILI and SARI cases were observed for Flu, SP, SARS-CoV-2, and RSV, while no significant differences were found for the remaining pathogens (P > 0.05).The top five pathogens detected in ILI cases were Flu (28.06%, 550/1960), SP (14.80%, 290/1960), RV (8.21%, 161/1960), SARS-CoV-2 (6.48%, 127/1960), and AdV (5.78%, 171/1960). The top five pathogens detected in SARI cases were Flu (11.90%, 100/840), SP (9.29%, 78/840), AdV and RV (tied for third, 7.62%, 64/840 each), MP (7.38%, 62/840), and RSV (5.12%, 43/840).The detection rates of Flu, SP, RV, HMPV, AdV, HCoV, EV, and SARS-CoV-2 were lower in SARI cases compared to ILI cases, whereas the detection rates of RSV, HPIV, HBoV, CP, and MP were higher in SARI cases than in ILI cases. Detailed comparisons are presented in Table 5.

**Table 5:** Comparison of pathogen detection rates between ILI and SARI cases [n (%)].

Pathogen	Total (n = 2800)	SARI (n = 840)	ILI (n = 1960)	χ²	*P*-value
Overall positive	1660(59.28)	398(47.38)	1262(64.39)	70.457	<0.001
Co-infections	439(15.68)	106(12.62)	333(16.99)	8.497	0.004
Flu	650(23.21)	100(11.90)	550(28.06)	86.106	<0.001
Influenza A	360(12.86)	47(5.60)	313(15.97)	56.481	<0.001
Influenza B	290(10.36)	53(6.31)	237(12.09)	21.175	<0.001
MP	174(6.21)	62(7.38)	112(5.71)	2.803	0.094
SP	368(13.14)	78(9.28)	290(14.80)	15.639	<0.001
SARS-CoV-2	153(5.46)	26(3.09)	127(6.48)	13.308	<0.001
RV	225(8.04)	64(7.62)	161(8.21)	0.282	0.595
HMPV	81(3.00)	22(2.62)	59(3.01)	0.320	0.571
ADV	235(8.39)	64(7.62)	171(8.72)	0.935	0.334
RSV	107(3.82)	43(5.12)	64(3.26)	5.498	0.019
HPIV	55(1.96)	20(2.38)	35(1.78)	1.082	0.298
HCoV	51(1.82)	15(1.78)	36(1.84)	0.009	0.926
HBoV	10(0.36)	4(0.48)	6(0.31)	0.119	0.730
CP	22(0.78)	9(1.07)	13(0.66)	1.257	0.262
EV	52(1.86)	12(1.43)	40(2.04)	1.209	0.271

(Note: Data are presented as n (%). Abbreviations are as in Table 3.)

### 3.5 Multivariable analysis

Multivariable logistic regression was performed to adjust for potential confounders. After controlling for age, sex, case type, month, and region, age ≤ 5 years (OR = 5.21, 95% CI: 3.79–7.15, P < 0.001), age 6–14 years (OR = 3.45, 95% CI: 2.51–4.74, P < 0.001), and age 15–64 years (OR = 1.98, 95% CI: 1.48–2.64, P < 0.001) remained significantly associated with higher positivity compared with the ≥ 65 years group. ILI cases had higher odds of positivity than SARI cases (OR = 1.92, 95% CI: 1.58–2.33, P < 0.001). Positivity decreased with each successive month (OR = 0.89 per month, 95% CI: 0.86–0.92, P < 0.001). Compared with Changshan County, Kaihua County (OR = 3.12, 95% CI: 2.21–4.40, P < 0.001), Kecheng District (OR = 2.45, 95% CI: 1.78–3.37, P < 0.001), Longyou County (OR = 2.32, 95% CI: 1.69–3.18, P < 0.001), and Qujiang District (OR = 1.58, 95% CI: 1.14–2.19, P = 0.006) showed significantly higher positivity; Jiangshan City was not significantly different (OR = 1.12, 95% CI: 0.79–1.58, P = 0.523). Sex was not an independent predictor (OR = 0.96, 95% CI: 0.82–1.12, P = 0.582). The Hosmer-Lemeshow test indicated good model fit (χ² = 8.24, P = 0.41).

## 4 Discussion

Acute respiratory infections (ARIs) are the fourth leading cause of death globally, imposing a substantial disease burden on public health worldwide [11]. The epidemiological patterns of respiratory pathogens have undergone significant changes in the context of the COVID-19 pandemic, with marked declines in the detection of common ARIs observed compared to other years [4]. The implementation of COVID-19 prevention and control measures reduced opportunities for exposure to respiratory pathogens, consequently leading to a diminished population-level immune barrier against common respiratory pathogens, particularly among young children. This immune naivety may have contributed to more severe circulation and transmission of respiratory pathogens following the conclusion of the pandemic [5]. The concept of “immunity debt” has been proposed to explain the resurgence of respiratory infections after lifting of non-pharmaceutical interventions [12]. Similarly, a global rebound of respiratory pathogens has been documented in multiple countries following the COVID-19 pandemic [13].In addition to the impact of non-pharmaceutical interventions and immunity debt, viral evolution—particularly the emergence of SARS-CoV-2 variants of concern (e.g., Alpha, Delta, Omicron)—has also played a critical role in shaping transmission dynamics and disease severity during the pandemic [14]. These variants have demonstrated altered transmissibility, immune evasion, and pathogenicity, which may have indirectly influenced the circulation patterns of other respiratory pathogens through viral interference or shifting population susceptibility. Future surveillance should integrate genomic monitoring to detect such evolutionary changes.The respiratory tract provides an ecological niche for the coexistence of multiple pathogens across temporal and spatial dimensions, potentially leading to interactions among different pathogens and resulting in changes to epidemiological patterns, including seasonality, activity intensity, and co-infection dynamics [2]. The year 2023 marked the first year in China when COVID-19 management was adjusted from Class A to Class B. Analysis of multi-pathogen surveillance data for acute respiratory infections in Quzhou City during 2023−2024 is conducive to understanding the epidemiological distribution characteristics of other respiratory pathogens in the Quzhou region after the COVID-19 pandemic, thereby providing a scientific basis for formulating subsequent respiratory infection prevention and control strategies.

The overall ARI positivity rate in our study (59.28%) was higher than that previously reported in Beijing (31.1%) [6] but lower than that reported in Ganzhou City in 2021 (61.11%) [7]. No statistically significant difference in pathogen positivity rates was observed between sexes (P = 0.545). However, children aged 5 years and below exhibited the highest positivity rate, while individuals aged ≥ 65 years showed the lowest positivity rate. This finding may be attributed to the implementation of a public health program in Quzhou City in recent years, providing free influenza vaccination to residents aged 60 years and above.During 2023–2024, the overall positivity rate of acute respiratory pathogens in Quzhou City showed a fluctuating trend, which is consistent with the seasonal epidemiological characteristics of respiratory infectious diseases. Beginning in November 2023, the monthly pathogen detection rate demonstrated an overall pattern of initial decrease, followed by an increase, and then another decrease, with inflection points occurring in February 2024 and April 2024. Furthermore, significant differences in respiratory multi-pathogen positivity rates were observed across surveillance regions, with Kaihua County showing the highest detection rate and Changshan County the lowest. This variation may be influenced by multiple factors, including population density, age structure, healthcare-seeking behavior, and true epidemiological differences.Regarding pathogen detection rates, ILI cases showed significantly higher positivity rates compared to SARI cases. It is speculated that this may be related to the fact that infections caused by most respiratory pathogens included in this study typically present with mild symptoms and a relatively low proportion of severe cases, in addition to the likelihood that SARI cases had already received treatment prior to sampling.

The top three pathogens detected in this study were influenza virus (Flu), Streptococcus pneumoniae (SP), and adenovirus (AdV). Pathogen positivity was predominantly characterized by single infections, with an overall co-infection detection rate of 15.68%. This was higher than the co-infection rate of 10.80% reported in a study from Tongzhou District, Beijing [8], but fell within the range of 0.60% to 27.00% reported for respiratory pathogen co-infection rates in domestic and international studies [15,16]. In 2023, the WHO issued a statement indicating that Mycoplasma pneumoniae (MP) is one of the pathogens contributing to the increasing number of pediatric community-acquired pneumonia (CAP) cases in China [17]. Streptococcus pneumoniae (SP), as an important opportunistic pathogen, primarily colonizes the nasopharynx of children and can cause various diseases when immune function declines [18]. This may explain the relatively high detection rates of SP and MP observed in individuals under 14 years of age in this study.Human rhinovirus (RV) is one of the major pathogens causing the common cold. Rhinovirus infection typically presents as a mild, self-limiting illness, with most common manifestations being upper respiratory symptoms such as nasal congestion, rhinorrhea, and sore throat. However, it can cause severe disease in infants, the elderly, and immunocompromised individuals [19]. Surveillance data from this study showed that rhinovirus was detected throughout the entire study period, suggesting that continuous surveillance of rhinovirus should be maintained.Human parainfluenza virus (HPIV) is also an important respiratory pathogen that can cause bronchitis and pneumonia in infants and immunocompromised populations, ranking as the second most common pathogen causing acute respiratory infections in children after respiratory syncytial virus [20]. In Quzhou, children aged 0–5 years were the most susceptible population for both respiratory syncytial virus and parainfluenza virus infections.Human metapneumovirus (HMPV) is widely prevalent worldwide, with high infection and mortality rates [3,21]. In this surveillance, relatively higher detection rates of HMPV were observed in children aged 0–5 years (4.76%) and individuals aged 6–14 years (3.37%), indicating the need to strengthen surveillance for HMPV across all age groups.The seasonality of respiratory viruses is a well-recognized phenomenon in the Northern Hemisphere. In China, influenza virus typically peaks during late autumn to early spring (November–March), which aligns with our observations of high Flu detection rates in November–February (up to 46.85%). Similarly, RSV is known to circulate predominantly in autumn and winter months in temperate regions, with peak activity often occurring between October and January [22]. In our study, RSV activity peaked in March (12.60%) and was also notable in children ≤5 years (12.38%), suggesting a slightly shifted but still winter-spring pattern. Understanding these seasonal patterns helps guide timing of vaccination campaigns and public health messaging.

This study has several limitations. First, the surveillance period covered only nine months (November 2023–July 2024), missing a full annual cycle; therefore, our descriptions of seasonal patterns are preliminary and require validation with complete year‑round data. Second, monthly case numbers were highly unbalanced (from 30 in July to 463 in January), which may affect the stability of rate estimates for months with small sample sizes. Third, the use of different specimen types among SARI cases (though mostly NP swabs) introduces potential heterogeneity in diagnostic sensitivity. Fourth, detection of Streptococcus pneumoniae by nasopharyngeal PCR cannot distinguish colonization from infection. Fifth, we lacked pre‑pandemic baseline data from the same population, so we cannot claim a true “shift” in the pathogen spectrum; instead we present a descriptive snapshot of the post‑relaxation period. Sixth, because no correction for multiple comparisons was applied, some significant findings may remain due to chance. Seventh, ethical approval was obtained after data collection had started; however, this is consistent with retrospective studies using pre‑existing, anonymized samples, and no analysis was performed before approval (data accessed on 15 Jan 2025). Eighth, we did not collect clinical outcome data. Consequently, we could not assess the association between specific pathogens (or co-infection patterns) and disease severity. Future studies incorporating clinical endpoints are needed to evaluate the clinical implications of the observed pathogen spectrum.Ninth, while we used a clinically validated multiplex PCR kit, we did not further confirm the positive results by sequencing or viral/bacterial isolation. PCR detects nucleic acids and cannot prove the presence of viable pathogens or distinguish colonization from infection for certain bacteria (e.g., SP). Future studies incorporating culture or sequencing would strengthen etiological confirmation.

## Supporting information

S1 Table Summary table of multipathogen nucleic acid testing across counties/cities/districts of Quzhou City, November 2023 to July 2024. (S1_Table (20260515).XLSX)

## References

[1] WHO. Global Health Estimates 2020: Deaths by Cause, Age, Sex, by Country and by Region, 2000-2019. https://www.who.int/data/gho/data/themes/mortality-and-global-health-estimates

[2] Ai J, Wang H, Zhang H, Song J, Zhang Y, Lin K, et al. Alterations of pathogen transmission patterns and attenuated immune stimulation might be the cause of increased adult respiratory infections cases in 2023, results from a multi-center study in mainland China. Heliyon. 2024;10(12):e32304. doi: 10.1016/j.heliyon.2024.e32304 38948033 PMC11209019

[3] Ma FL, Zheng LS. Research progress on human metapneumovirus infection. Chin J Virol. 2024;40(1):133–9. doi: 10.13242/j.cnki.bingduxuebao.004438

[4] Tang JW, Bialasiewicz S, Dwyer DE, Dilcher M, Tellier R, Taylor J, et al. Where have all the viruses gone? Disappearance of seasonal respiratory viruses during the COVID-19 pandemic. J Med Virol. 2021;93(7):4099–101. doi: 10.1002/jmv.26964 33760278 PMC8250511

[5] Chu YR, Lei S, Lao XY. Epidemiological characteristics of multi-pathogen in acute respiratory infection cases in Ningbo during winter and spring of 2023-2024. Mod Pract Med. 2024;36(9):1162–5. doi: 10.3969/j.issn.1671-0800.2024.09.012

[6] Qin JN, Chu YH, Sun JY. Epidemiological characteristics of 11 pathogens in respiratory infection cases in Xicheng District, Beijing, 2014-2020. Int J Virol. 2022;29(1):18–22. doi: 10.3760/cma.j.issn.1673-4092.2022.01.004

[7] Hu XJ, Su JJ, Yan FY. Multi-pathogen study of respiratory cases with fever symptoms in Ganzhou City. Contemp Med. 2023;30(25):43–7. doi: 10.3969/j.issn.1674-4721.2023.25.011

[8] Zou L, Gao X, Zhang C. Epidemiological characteristics of respiratory pathogens in patients with respiratory infection in Tongzhou District, Beijing, 2020-2022. Disease Surveillance. 2023;38(7):799–805. doi: 10.3784/jbjc.202303220122

[9] National Health and Family Planning Commission of the People’s Republic of China. Notice of the General Office of the National Health and Family Planning Commission on Issuing the National Influenza Surveillance Protocol (2017 Version). [cited 2024 May 1]. http://www.nhc.gov.cn/jkj/s3577/201704/ed1498d9e64144738cc7f8db61a39506.shtml

[10] Liu BM. Isothermal Nucleic Acid Amplification Technologies and CRISPR‐Cas‐Based Nucleic Acid Detection Strategies for Infectious Diseases Diagnostics. Manual of Molecular Microbiology. Wiley. 2025. p. 30–47. doi: 10.1002/9781683674597.ch3

[11] Wang HR, Mou JB, Qiu Q. Analysis of surveillance results for viral acute respiratory infections in Shanghai, 2023. Chin J Exp Clin Virol. 2024;38(4):439–45. doi: 10.3760/cma.j.cn112866-20240409-00058

[12] Cohen R, Ashman M, Taha M-K, Varon E, Angoulvant F, Levy C, et al. Pediatric Infectious Disease Group (GPIP) position paper on the immune debt of the COVID-19 pandemic in childhood, how can we fill the immunity gap?. Infect Dis Now. 2021;51(5):418–23. doi: 10.1016/j.idnow.2021.05.004 33991720 PMC8114587

[13] Olsen SJ, Winn AK, Budd AP. Changes in influenza and other respiratory virus activity during the COVID-19 pandemic-United States, 2020-2021. Am J Transplant. 2021;21(10):3481–6. doi: 10.1111/ajt.16049 34624182 PMC8653380

[14] Liu BM, Yao Q, Cruz-Cosme R, Yarbrough C, Draper K, Suslovic W, et al. Genetic conservation and diversity of SARS-CoV-2 envelope gene across variants of concern. J Med Virol. 2025;97(1):e70136. doi: 10.1002/jmv.70136 39744807 PMC12228529

[15] Kabir MS, Clements MO, Atkins M, Kimmitt PT. Application of RT-Bst to enhance detection of pathogenic viruses of the respiratory tract. Br J Biomed Sci. 2015;72(3):128–34. doi: 10.1080/09674845.2015.11666809 26510269

[16] Cao YW, Shang XY, Tang XP. Etiological study of common respiratory viruses in adults with acute respiratory infection. Military Medical Sciences. 2014;38(1):74–6. doi: 10.7644/j.issn.1674-9960.2014.01.020

[17] Li H, Li S, Yang H, Chen Z, Zhou Z. Resurgence of Mycoplasma pneumonia by macrolide-resistant epidemic clones in China. Lancet Microbe. 2024;5(6):e515. doi: 10.1016/S2666-5247(23)00405-6 38244553

[18] Chen Y, Deng W, Wang S-M, Mo Q-M, Jia H, Wang Q, et al. Burden of pneumonia and meningitis caused by Streptococcus pneumoniae in China among children under 5 years of age: a systematic literature review. PLoS One. 2011;6(11):e27333. doi: 10.1371/journal.pone.0027333 22110628 PMC3217934

[19] Jacobs SE, Lamson DM, George KS. Human rhinoviruses. Clin Microbiol Rev. 2013;26(1):135–62. doi: 10.1128/CMR.00077-12 23297263 PMC3553670

[20] Feng L, Li Z, Zhao S. Viral etiologies of hospitalized acute lower respiratory infection patients in China, 2009-2013. PLoS ONE. 2014;9(6):e99419. doi: 10.1371/journal.pone.0099419 PMC4063718 24945280

[21] Jain S, Self WH, Wunderink RG, Fakhran S, Balk R, Bramley AM, et al. Community-acquired pneumonia requiring hospitalization among U.S. Adults. N Engl J Med. 2015;373(5):415–27. doi: 10.1056/NEJMoa1500245 26172429 PMC4728150

[22] Contes KM, Liu BM. Epidemiology, clinical significance, and diagnosis of respiratory viruses and their co-Infections in the post-COVID era. Pathogens. 2025;14(3):262. doi: 10.3390/pathogens14030262 40137747 PMC11944763

