# Supplementary material for: Pathogen spectrum and epidemiology of respiratory tract infections in Quzhou, Eastern China, from November 2023 to July 2024: A post‑COVID‑19 surveillance study
Source: PLoS One. 2026 Jun 8;21(6):e0346441. doi: 10.1371/journal.pone.0346441 (PMC13245782; doi:10.1371/journal.pone.0346441)
Supplement: Attachment — Submitted filename: Comments-PlosOne.pdf [file pone.0346441.s002.pdf]

Manuscript ID: PONE-D-26-11954 **Title:** *Shifts in the pathogen spectrum and epidemiology of respiratory tract infections in the post-COVID-19 era: A study from Quzhou, Eastern China*

SUMMARY

Study presents surveillance data on 13 respiratory pathogens detected by multiplex real-time PCR from 2,800 ARI cases (ILI and SARI) collected in Quzhou City, Zhejiang Province, China, between November 2023 and July 2024. The authors report an overall positivity rate of 59.28%, with influenza virus, *Streptococcus pneumoniae*, and adenovirus as the top three pathogens. They describe age-specific, temporal, and geographic distributions and a co-infection rate of 15.68%. The topic is timely and relevant. However, the manuscript has several significant methodological, analytical, and presentation shortcomings that must be addressed before it can be considered for publication.

OVERALL RECOMMENDATION: Major Revision Required

MAJOR CONCERNS

1. Study Design and Sampling — Critical Methodological Weakness

1.1 Justification for sample size. The manuscript does not describe how the 2,800 cases were selected. Does all eligible ARI cases during the study period enrolled, or was there a sampling strategy? There is no sample size calculation or power analysis to justify the 1,960 ILI : 840 SARI ratio. Without this, it is unclear whether the proportions reflect true disease burden or selection bias. The authors must clarify the enrollment process in detail.

1.2 Marked temporal imbalance in monthly case numbers. Monthly case numbers varies dramatically — from just 30 cases in July to 463 in January. This extreme imbalance severely limits the validity of temporal comparisons, particularly for the months of June (n=90) and July (n=30). The 73.33% positivity rate in July, based on only 30 cases, is statistically unreliable and should not be interpreted alongside months with hundreds of cases. The authors must either acknowledge this as a critical limitation prominently, exclude under-powered monthly data from trend analysis, or apply appropriate corrections for unequal group sizes.

1.3 ILI and SARI case definitions are inconsistent with their claimed source. The ILI definition used (fever ≥38°C + cough or sore throat) and SARI definition (fever ≥38°C + cough or sore throat, onset within 10 days) are essentially clinically indistinguishable in their written form. The differentiation is described as outpatient vs. inpatient setting, is not a clinical severity variable. The WHO SARI case definition requires lower respiratory tract involvement (e.g., shortness of breath). The authors should clarify whether clinical severity criteria beyond setting were applied, and reconcile their definitions with the cited Zhejiang Provincial Protocol and WHO standards.

1.4 The study period covers only 9 months (November 2023–July 2024), missing the summer–autumn respiratory season entirely. I think this is limitation for a study claiming to characterize

post-COVID-19 epidemiological shifts. The authors do acknowledge this in the limitations section, but the impact is underplayed. Claims about "seasonal patterns" are not valid unless it is complete annual cycle data. The Discussion should be substantially restrained in its seasonal interpretations.

2. Laboratory Methods — Needs More Detail for Reproducibility

2.1 No internal controls or quality assurance described. The manuscript states that the Jiangsu Bioperfectus 13-pathogen PCR kit was used, but provides no information on: positive/negative extraction controls, inter-run variability, Ct value thresholds used to define a positive result, or validation of the kit against reference standards. For a surveillance study where all conclusions are based entirely on PCR results, this is a critical omission.

2.2 Specimen type heterogeneity is not addressed analytically. For SARI cases, specimens included throat swabs, nasal swabs, nasopharyngeal swabs, bronchoalveolar lavage fluid (BALF), and pleural puncture fluid. These specimen types have substantially different diagnostic sensitivities for different pathogens — for example, BALF is far more sensitive for lower respiratory pathogens like RSV and MP than a throat swab. No stratification by specimen type is presented. This is particularly important for the ILI vs. SARI comparison, where the apparent differences in pathogen detection rates may simply reflect specimen type differences rather than true clinical differences. The authors must either stratify by specimen type or explicitly justify why this was not done, and acknowledge this as a significant potential confounder.

2.3 *Streptococcus pneumoniae* detection by PCR requires important caveats. SP detection by nasopharyngeal/throat swab PCR cannot distinguish colonization from true infection. The authors acknowledge SP as an "opportunistic pathogen that colonizes the nasopharynx" in the Discussion but then present SP detection rates alongside viral pathogens without qualifying this distinction in the Results. A detection rate of 13.14% for SP, presented equivalently to influenza virus, is potentially misleading. The authors should explicitly state this limitation in both the Methods and Results sections, and discuss its implications for interpreting the true infectious burden attributable to the pathogen.

3. Statistical Analysis — Incomplete and Partially Inappropriate

3.1 The manuscript uses only χ^2 tests and a single trend test (for age vs. co-infection). Given that age, season, case type, and geographic region are highly interrelated variables and multivariable analysis would substantially strengthen the study. At minimum, a logistic regression model examining predictors of pathogen positivity or co-infection — adjusting for age, sex, case type, and month — would substantially strengthen the study.

3.2 The co-infection rate of 15.68% (26.45% of positive cases) is a central finding, yet the specific pathogen pairs or combinations involved are never reported. Which pathogen combinations were most common? Were certain combinations associated with ILI vs. SARI, or with specific age groups? This is a missed opportunity for meaningful analysis that the multiplex PCR data would allow, and is important given the stated interest in co-infection dynamics.

3.3 No correction for multiple comparisons. Thirteen pathogens are each individually tested across multiple demographic and clinical subgroups (4 age groups, 6 regions, 2 case types, 9 months), generating a very large number of statistical comparisons. No Bonferroni or equivalent correction is applied or even discussed. Some of the reported statistically significant findings (particularly for rare pathogens such as HBoV, CP, HCoV) may not survive correction and should be interpreted with caution.

3.4 Trend analysis for age and co-infection lacks detail. A Cochran-Armitage trend test is mentioned but the result is only briefly noted (χ^2=172.511, P<0.01) for the overall positivity-age relationship. The actual trend test for co-infection rate vs. age — which is stated as a method — does not appear in the Results tables or narrative. Its outcome should be explicitly reported.

4. Ethical and Data Concerns

4.1 Critical inconsistency in informed consent disclosure. The manuscript submission form states: "*Written informed consent was obtained from all individual participants included in the study. For participants under 18 years of age, written informed consent was obtained from their parent or legal guardian*."

However, the Methods section of the manuscript states that the study used *retrospective residual specimens* and that *informed consent was waived* by the ethics committee due to the retrospective and anonymized nature of the study.

4.2 Ethics approval date post-dates data collection. The ethics approval date is stated as 24 September 2024, while data collection ran from November 2023 to July 2024. While retrospective approval is not uncommon, this must be explicitly acknowledged and justified in the Ethics Statement. The current manuscript does note "data were accessed for research purposes on 15/01/2025," but this context needs to be more transparent to ensure the ethics committee had full knowledge of the retrospective scope.

4.3 Data availability statement is contradictory. The submission form declares data are "fully available without restriction," but the manuscript itself states data are "available from the corresponding author upon reasonable request."

5. Scope and Generalizability — Overstated Claims

5.1 The title and framing overstates the study's conclusions. The title claims to characterize "shifts in the pathogen spectrum...in the post-COVID-19 era." However, the study has no pre-pandemic or pandemic-era comparison data from the same population. Without a baseline from Quzhou itself, the study can only describe the current pathogen distribution — it cannot demonstrate a "shift." Comparisons with other cities (Beijing, Ganzhou) are insufficient to establish a temporal shift in Quzhou specifically. The authors should either reframe the title and objectives to accurately reflect a descriptive surveillance study, or obtain and present comparative pre-pandemic data from the same surveillance network.

5.2 The claim that geographic variation reflects "differences in professional expertise" is unsubstantiated. The authors attribute the variation in positivity rates between counties (40.55% to 72.47%) to differences in medical staff sampling practices. This is speculative and not supported by any data presented. Other explanations — population density, age distribution, healthcare-seeking behavior, true epidemiological differences — are equally plausible and should be acknowledged.

MINOR CONCERNS

6.1 Language and formatting issues.

- Table 1 in the main manuscript contains Chinese characters for sex categories, which must be replaced with English throughout. This suggests a version control error between the Chinese and English manuscripts.
- Abbreviations are inconsistently capitalized (e.g., "AdV" vs. "ADV," "Flu" vs. "FLU") across tables and text. A consistent convention should be established and applied throughout.

6.2 The following must be addressed as mandatory revisions:

1. Resolve the contradiction in informed consent disclosures and clarify the ethics timeline
2. Correct the data availability statement and deposit data in a public repository
3. Provide sample size justification and describe the enrollment/sampling strategy
4. Address specimen type heterogeneity in the SARI group analytically or acknowledge it explicitly as a major confounder
5. Add Ct value thresholds and quality control details for the PCR assay
6. Provide specific co-infection pair/combination data
7. Perform multivariable analysis or provide explicit justification for why it was not done
8. Revise the title and objectives to accurately reflect the descriptive cross-sectional nature of the study
9. Correct all Chinese-language text in tables and formatting artifacts
10. Add percentage-based detection rates to Table 4
11. Include meaningful figures (temporal trends, age distribution, geographic map) ibution, unlike Tables 3 and 5 which provide both counts and percentages. Given the highly unequal monthly case numbers, raw counts alone are misleading. Detection rates (%) should be added for each pathogen per month.

6.3 No figures are present in the manuscript. For a surveillance and epidemiology study of this nature, visual representations are essential for clarity. The authors should include: (a) a figure showing monthly positivity rates and dominant pathogens over the study period, (b) an age-stratified pathogen distribution figure, and (c) a geographic map showing positivity rates across the six counties.

6.4 The Discussion is largely descriptive and lacks depth. The Discussion mostly restates Results and makes comparisons with a small number of regional Chinese studies. There is inadequate engagement with the international literature on post-pandemic respiratory pathogen dynamics, immunity debt, the epidemiology of mixed infections, or the clinical implications of specific

pathogen combinations. The authors should substantially expand the Discussion to contextualize findings within the broader global post-COVID-19 respiratory surveillance literature.

6.5 Reference quality and formatting. Several references are Chinese-language journal articles or grey literature with limited international accessibility. While these may be appropriate for local context, the authors should ensure that key epidemiological claims are supported by internationally indexed literature. Reference [2] (a thesis/dissertation) should be used cautiously and supplemented with peer-reviewed sources. Reference formatting should be checked for consistency throughout.

6.6 The clinical relevance and implications of findings are underdeveloped. The study is based at a Center for Disease Control and Prevention but provides no clinical correlation data — no information on severity outcomes, hospitalization duration, treatment requirements, or mortality across pathogen types. Even basic clinical outcome data stratified by pathogen or co-infection status would substantially increase the manuscript's clinical value and relevance to PLOS ONE's readership.

SUMMARY OF REQUIRED REVISIONS

The following must be addressed as mandatory revisions:

1. Resolve the contradiction in informed consent disclosures and clarify the ethics timeline
2. Correct the data availability statement and deposit data in a public repository
3. Provide sample size justification and describe the enrollment/sampling strategy
4. Address specimen type heterogeneity in the SARI group analytically or acknowledge it explicitly as a major confounder
5. Add Ct value thresholds and quality control details for the PCR assay
6. Provide specific co-infection pair/combination data
7. Perform multivariable analysis or provide explicit justification for why it was not done
8. Revise the title and objectives to accurately reflect the descriptive cross-sectional nature of the study
9. Correct all Chinese-language text in tables and formatting artifacts
10. Add percentage-based detection rates to Table 4
11. Include meaningful figures (temporal trends, age distribution, geographic map)

This review reflects the independent scientific assessment by me and I have no conflicts of interest with respect to this manuscript.
