# Supplementary material for: Pathogen spectrum and epidemiology of respiratory tract infections in Quzhou, Eastern China, from November 2023 to July 2024: A post‑COVID‑19 surveillance study
Source: PLoS One. 2026 Jun 8;21(6):e0346441. doi: 10.1371/journal.pone.0346441 (PMC13245782; doi:10.1371/journal.pone.0346441)
Supplement: Attachment — Submitted filename: Response to Reviews(20260515).docx [file pone.0346441.s004.docx]

Response to Reviewers

Manuscript ID: PONE-D-26-11954
Title: Shifts in the pathogen spectrum and epidemiology of respiratory tract infections in the post-COVID-19 era: A study from Quzhou, Eastern China (revised title: Pathogen spectrum and epidemiology of respiratory tract infections in Quzhou, Eastern China, from November 2023 to July 2024: a post‑COVID‑19 surveillance study)

Dear Dr. Benjamin M. Liu and the Reviewers,

We sincerely thank the Academic Editor and the reviewers for their careful reading of our manuscript and for their constructive comments. We have revised the manuscript thoroughly in response to all the points raised. Below we provide a point‑by‑point response.

Responses to the Academic Editor’s comments

Comment 1:
The authors stated that "Respiratory specimens used in this study were residual samples collected for routine clinical care purposes between November 2023 and July 2024." This time window covered typical respiratory season in China. Some respiratory viruses, e.g., RSV and flu, have seasonality and prevalence in fall/winter in China. The authors should discuss seasonality and prevalence of these pathogens in fall/winter in northern hemisphere. More references should be cited, with this one (PMID: 40137747) as an example.

Response:
We agree and have expanded the Discussion to address the seasonality of respiratory viruses in the Northern Hemisphere, with a specific focus on influenza and RSV. We now explicitly state that in China, influenza typically peaks from late autumn to early spring (November–March), which aligns with our observations. RSV predominates in autumn and winter in temperate regions. We have cited the recommended reference (Contes & Liu, Pathogens 2025, PMID: 40137747) and added a new reference [22] accordingly.
Location: Discussion, fourth paragraph.

Comment 2:
The authors should introduce or discuss that PCR is just one type (i.e., thermal NAAT) of NAATs for these pathogens and there are isothermal NAATs that are widely used. More references should be cited, with the Liu BM book chapter as an example.

Response:
We have added a brief discussion in the Methods section (subsection 2.3) to clarify that conventional PCR is a thermal‑cycling‑based nucleic acid amplification test (NAAT) and that isothermal NAATs (e.g., LAMP, RPA) are also widely used for rapid detection of respiratory pathogens. The recommended reference (Liu BM, Manual of Molecular Microbiology, ASM Press, 2025) has been cited as reference [10].
Location: Methods, section 2.3, last sentence.

Comment 3:
The authors focused on preventive procedures. Besides, the authors should introduce the impact of viral evolution, e.g., SARS-CoV-2 variants of concern, on the transmission and disease severity. More references should be cited, with this one (PMID: 39744807) as an example.

Response:
We have added a new paragraph in the Discussion (third paragraph) addressing the impact of SARS‑CoV‑2 variants of concern (Alpha, Delta, Omicron) on transmissibility, immune evasion, and disease severity, and how these changes might have indirectly influenced the circulation of other respiratory pathogens. The recommended reference (Liu BM et al., J Med Virol 2025, PMID: 39744807) is now cited as reference [14].
Location: Discussion, third paragraph.

Responses to Reviewer #2

We thank Reviewer #2 for the detailed and constructive critique. All mandatory revisions have been addressed, as detailed below.

Mandatory revision 1 – Resolve the contradiction in informed consent disclosures and clarify the ethics timeline
We have completely rewritten the Ethics Statement (Methods, section 1) to resolve the inconsistency. The study was retrospective and used fully anonymized residual specimens; therefore, the ethics committee waived the requirement for written informed consent. We also explain that ethical approval (24 September 2024) was obtained after the start of data collection (November 2023) but before any analysis was performed (data were accessed for research on 15 January 2025). This is standard for retrospective studies using pre‑existing, de‑identified data.
Location: Methods, section 1 (Ethics statement).

Mandatory revision 2 – Correct the data availability statement and deposit data in a public repository.

We have revised the Data Availability Statement to fully comply with PLOS ONE’s data policy. The anonymized aggregate data supporting the findings of this study are now provided in Supporting Information S1 Table, which has been uploaded as a supplementary file. Patient-level data cannot be publicly shared due to patient privacy protection. De-identified individual data may be available from the corresponding author upon reasonable request, subject to approval by the institutional ethics committee. This statement has been included at the end of the manuscript (Data Availability Statement section).

Location: Data Availability Statement section.

Mandatory revision 3 – Provide sample size justification and describe the enrollment/sampling strategy
We have added a detailed description in Methods 2.1. All consecutive ARI patients meeting the case definitions at the participating county‑level hospitals during the study period were included; no additional sampling or selection was applied. The ILI:SARI ratio (1,960:840) reflects the natural distribution in our surveillance network. A post‑hoc power analysis shows that with 2,800 cases, the study had 84% power to detect a 10% absolute difference in positivity rates between subgroups (α=0.05, two‑sided).
Location: Methods, section 2.1, paragraphs 2–3.

Mandatory revision 4 – Address specimen type heterogeneity in the SARI group analytically or acknowledge it explicitly as a major confounder
We have added a new subsection 2.5 in Methods to describe the specimen types:among SARI cases, the majority (83.6%, 702/840) were throat swabs; bronchoalveolar lavage fluid (BALF, n=130) and pleural fluid (n=8) were used in a minority. Given the small number of non‑throat swab specimens (n=138, 16.4%), formal statistical comparison was not performed.

Location: Methods, section 2.5; and Limitations (Discussion, third point).

Mandatory revision 5 – Add Ct value thresholds and quality control details for the PCR assay
We have added a new subsection 2.4 in Methods that describes: a sample as positive when Ct <35; Ct values 35–38 were re‑tested and considered borderline positive if still <38; inclusion of positive/negative extraction controls, no‑template control, and no‑reverse‑transcriptase control for RNA pathogens; assessment of inter‑run variability (CV <5% for all targets).
Location: Methods, section 2.4.

Mandatory revision 6 – Provide specific co‑infection pair/combination data
We have added detailed co‑infection data in the Results (section 2.2, within the parentheses). For dual infections, the most frequent combinations were RV+SP, AdV+SP, and RSV+SP. For triple infections, SARS‑CoV‑2+RV+SP and SP+RV+AdV predominated. Quadruple and higher‑order co‑infections were also observed, with examples provided.
Location: Results, section 2.2, after Table 2.

Mandatory revision 7 – Perform multivariable analysis or provide explicit justification for why it was not done
We have performed a multivariable binary logistic regression analysis. The dependent variable was overall positivity; independent variables included age group, sex, case type, month, and region. Adjusted odds ratios with 95% CIs are reported. Age ≤5 years, ILI cases, and certain regions (Kaihua, Kecheng, Longyou, Qujiang) were independent predictors of higher positivity. The Hosmer‑Lemeshow test indicated good model fit (χ²=8.24, P=0.41). This is now presented in Results section 5 and described in Methods section 4.
Location: Methods, section 4 (Statistical Analysis); Results, section 5 (Multivariable analysis).

Mandatory revision 8 – Revise the title and objectives to accurately reflect the descriptive cross‑sectional nature of the study
We have revised the title to: "Pathogen spectrum and epidemiology of respiratory tract infections in Quzhou, Eastern China, from November 2023 to July 2024: a post‑COVID‑19 surveillance study" The objectives in the Abstract and Introduction have been rephrased to reflect a descriptive surveillance study rather than claiming to demonstrate a "shift" without local pre‑pandemic data.
Location: Title page; Abstract, Objective; Introduction, last paragraph.

Mandatory revision 9 – Correct all Chinese‑language text in tables and formatting artifacts
All Chinese characters have been removed from tables. Sex categories are now "Male/Female". Abbreviations are consistent (e.g., AdV, Flu, MP, SP). Table formatting has been standardized.
Location: Tables 1–5.

Mandatory revision 10 – Add percentage‑based detection rates to Table 4
Table 4 now presents both counts and percentages for each pathogen per month. For example, "46.18%" is shown alongside the absolute number.
Location: Table 4.

Mandatory revision 11 – Include meaningful figures (temporal trends, age distribution, geographic map)
We have added two figures: Figure 1 (positivity rates of common pathogens in different age groups) and Figure 2 (monthly positivity trends of major pathogens from November 2023 to July 2024). Due to the regulatory requirement in China that administrative boundary maps at the county/district level must undergo official review and approval prior to publication, a geographic map is not included in this manuscript. Moreover, given that our geographic analysis involves only six discrete counties/districts with clear numerical differences, a choropleth map would not add substantial information beyond the detailed numerical comparisons already presented in Results section 3.3. Therefore, we report the geographic distribution data in tabular form with explicit positivity rates for each region.

Location: Results, sections 3.1 and 3.2; Figures 1 and 2.

Additional major concerns raised by Reviewer #2 – we have also addressed the following:

· 1.2 Marked temporal imbalance (July n=30): We have now explicitly added a sentence in the Limitations section acknowledging that the small sample size in July (n=30) limits the reliability of the positivity rate estimate for that month.
 Location: Discussion, Limitations (second point).
· 1.3 ILI/SARI definitions: We have revised the SARI definition to include clinical evidence of lower respiratory tract involvement (shortness of breath, tachypnea, abnormal breath sounds) following WHO and Zhejiang Provincial Protocol.
 Location: Methods, section 2.1.
· 1.4 Missing summer‑autumn season: We clearly state in the Limitations that the study period covered only nine months and therefore seasonal descriptions are preliminary and require full annual data.
 Location: Discussion, Limitations (first point).
· 2.3 SP detection – colonization vs. infection: We added a cautionary note in Results (section 2.2) and in the Limitations that SP detection by nasopharyngeal PCR cannot distinguish colonization from infection and may overestimate the true infectious burden.
 Location: Results, section 2.2 (parentheses after SP data); Discussion, Limitations.
· 3.3 Multiple comparisons: We have added a sentence in the Statistical Analysis section that, given the exploratory nature of this study, no correction for multiple comparisons was applied; findings with borderline P values should be interpreted cautiously.
 Location: Methods, section 4, last sentence.
· 3.4 Trend test for co‑infection vs. age: The Cochran‑Armitage trend test result (χ²=41.2, P<0.001) is now explicitly reported in Results section 3.1, showing that the co‑infection rate decreased significantly with age.
 Location: Results, section 3.1, last sentence before Table 3.
· 5.2 Geographic variation – unsupported speculation: We have removed the speculative statement about "differences in professional expertise". We now simply report the observed differences and note that multiple factors (population density, age structure, healthcare‑seeking behavior, true epidemiological differences) may contribute.
 Location: Results, section 3.3; Discussion has no such speculation.
· 6.4 Discussion depth: We have substantially expanded the Discussion to include international literature on post‑pandemic respiratory pathogen dynamics, immunity debt, viral evolution, and the seasonality of respiratory viruses. Additional references have been added.
 Location: Discussion, entire section.
· 6.6 Lack of clinical outcome data: We have explicitly stated in Methods (2.1) that clinical outcome data (hospitalization duration, ICU admission, mortality) were not collected, and we have listed this as a limitation in the Discussion.
 Location: Methods, section 2.1, last sentence; Discussion, Limitations (eighth point).

Responses to Reviewer #3

Comment:
Authors reported the presence of multiple respiratory pathogens post‑COVID‑19 by Real‑time PCR. However, the MS did not confirm the presence of these pathogens either by sequencing or by isolation of these pathogens.

Response:
We thank the reviewer for this important point. We have added a specific limitation in the Discussion (ninth point) acknowledging that we did not further confirm positive PCR results by sequencing or culture. PCR detects nucleic acids and does not prove the presence of viable pathogens or distinguish colonization from infection for certain bacteria (e.g., S. pneumoniae). Future studies should incorporate culture or sequencing for stronger etiological confirmation.
Location: Discussion, Limitations (ninth point).

Responses to Reviewer #1

Reviewer #1 provided positive feedback with no specific revision requests. We thank the reviewer for the encouraging comments and for recognizing the value of our surveillance data.

Additional revisions made to comply with journal requirements

1. PLOS ONE style requirements: The manuscript has been formatted according to the PLOS ONE templates. All file naming will follow journal guidelines upon submission.
2. Data availability statement: Revised as described above.
3. Ethics approval timeline: Fully clarified in the Ethics Statement.
4. ORCID iD: The corresponding authors have verified their ORCID iDs.
5.Data availability and supplementary file: We have compiled all anonymized aggregate data into Supporting Information S1 Table and submitted it. The Data Availability Statement has been updated to indicate that the aggregate data are fully available in this supplementary file, while patient‑level data remain restricted to protect patient privacy.

Summary of changes

· Title modified to reflect descriptive surveillance.
· Methods expanded with specimen type breakdown, Ct thresholds, quality control, sample size justification, and multivariable analysis.
· Results updated with co‑infection pair data, trend test results, and multivariable regression.
· Tables 1–5 cleaned of Chinese characters and formatting errors; Table 4 now includes percentages.
· Figures 1 and 2 added.
· Discussion substantially rewritten to address seasonality, viral evolution, immunity debt, and limitations including small July sample, lack of pre‑pandemic baseline, and absence of sequencing/isolation confirmation.
· Ethical and data availability statements corrected.

We believe the manuscript has been substantially improved and now meets PLOS ONE’s publication criteria. We thank the editor and reviewers again for their time and valuable guidance.

We look forward to your favorable decision.

Sincerely,

Rui-jun Yang, Min Wang, Shi-teng Huang, Bing-dong Zhan (on behalf of all authors)
