# Supplementary material for: Pathogen spectrum and epidemiology of respiratory tract infections in Quzhou, Eastern China, from November 2023 to July 2024: A post‑COVID‑19 surveillance study
Source: PLoS One. 2026 Jun 8;21(6):e0346441. doi: 10.1371/journal.pone.0346441 (PMC13245782; doi:10.1371/journal.pone.0346441)
Supplement: Peer review history

# Peer review: Decision Letter 0

**DOI:** 10.1371/journal.pone.0346441.r001

**Authors:** Benjamin Liu

15 Apr 2026

PONE-D-26-11954 Shifts in the pathogen spectrum and epidemiology of respiratory tract infections in the post-COVID-19 era: A study from Quzhou, Eastern China PLOS One

Dear Dr. Yang,

Thank you for submitting your manuscript to PLOS ONE. After careful consideration, we feel that it has merit but does not fully meet PLOS ONE’s publication criteria as it currently stands. Therefore, we invite you to submit a revised version of the manuscript that addresses the points raised during the review process.

**ACADEMIC EDITOR'S COMMENTS:**1. The authors stated that "Respiratory specimens used in this study were residual samples collected for routine clinical care purposes between November 2023 and July 2024." This time window covered typical respiratory season in China. Some respiratory viruses, e.g., RSV and flu, have seasonality and prevalence in fall/winter in China. The authors should discuss seasonality and prevalence of these pathogens in fall/winter in northern hemisphere.  More references should be cited, with this one (PMID: 40137747) as an example (citing is optional).

2. The authors stated that " ABI real-time fluorescent PCR instruments and a ZY Biotechnology nucleic acid extraction system were used. PCR detection was performed using a 13-pathogen nucleic acid detection kit (Fluorescence PCR method) manufactured by Jiangsu Bioperfectus Technologies Co., Ltd. Specimens were transported to the virology laboratory at 4°C immediately after collection. Real-time RT-PCR was performed promptly to detect nucleic acids of influenza virus (Flu), Mycoplasma pneumoniae (MP), Streptococcus pneumoniae (SP), SARS-CoV-2, rhinovirus (RV), human metapneumovirus (HMPV), human adenovirus (AdV), respiratory syncytial virus (RSV), human parainfluenza virus (HPIV), human coronavirus (HCoV), human bocavirus (HBoV), Chlamydia pneumoniae (CP), and enterovirus (EV)." The authors should introduce or discuss that PCR is just one type (ie, thermal NAAT) of NAATs for these pathogens and there are isothermal NAATs that are widely used for the molecular testing of some of the pathogens of interest.  More references should be cited, with this one (Liu, B.M. Isothermal nucleic acid amplification technologies and CRISPR-Cas based nucleic acid detection strategies for infectious disease diagnostics. p 30-47. In Manual of Molecular Microbiology: Fundamentals and Applications; ASM Press: Washington, DC, USA, 2025.doi:10.1128/9781683674597.ch03.) as an example (citing is optional).

3. The authors stated that " The implementation of COVID-19 prevention and control measures reduced opportunities for exposure to respiratory pathogens, consequently leading to a diminished population-level immune barrier against common respiratory pathogens, particularly among young children." The authors focused on preventive procedures, which is important. Besides, the authors should introduce the impact of viral evolution, e.g., SARS-CoV-2 variants of concern, on the transmission and disease severity. More references should be cited, with this one (PMID: 39744807) as an example (citing is optional).

Please submit your revised manuscript by May 30 2026 11:59PM. If you will need more time than this to complete your revisions, please reply to this message or contact the journal office at plosone@plos.org. When you're ready to submit your revision, log on to https://www.editorialmanager.com/pone/ and select the 'Submissions Needing Revision' folder to locate your manuscript file.

Please include the following items when submitting your revised manuscript:

- A letter that responds to each point raised by the academic editor and reviewer(s). You should upload this letter as a separate file labeled 'Response to Reviewers'.
- A marked-up copy of your manuscript that highlights changes made to the original version. You should upload this as a separate file labeled 'Revised Manuscript with Track Changes'.
- An unmarked version of your revised paper without tracked changes. You should upload this as a separate file labeled 'Manuscript'.

If you would like to make changes to your financial disclosure, please include your updated statement in your cover letter. Guidelines for resubmitting your figure files are available below the reviewer comments at the end of this letter.

If applicable, we recommend that you deposit your laboratory protocols in protocols.io to enhance the reproducibility of your results. Protocols.io assigns your protocol its own identifier (DOI) so that it can be cited independently in the future. For instructions see: https://journals.plos.org/plosone/s/submission-guidelines#loc-laboratory-protocols. Additionally, PLOS ONE offers an option for publishing peer-reviewed Lab Protocol articles, which describe protocols hosted on protocols.io. Read more information on sharing protocols at https://plos.org/protocols?utm_medium=editorial-email&utm_source=authorletters&utm_campaign=protocols.

As the corresponding author, your ORCID iD is verified in the submission system and will appear in the published article. PLOS supports the use of ORCID, and we encourage all coauthors to register for an ORCID iD and use it as well. Please encourage your coauthors to verify their ORCID iD within the submission system before final acceptance, as unverified ORCID iDs will not appear in the published article. *Only* the individual author can complete the verification step; PLOS staff *cannot* verify ORCID iDs on behalf of authors.

We look forward to receiving your revised manuscript.

Kind regards,

Benjamin M. Liu, MBBS, PhD, D(ABMM), MB(ASCP)

Academic Editor

PLOS One

Journal requirements:

When submitting your revision, we need you to address these additional requirements.

1. Please ensure that your manuscript meets PLOS ONE's style requirements, including those for file naming. The PLOS ONE style templates can be found at

https://journals.plos.org/plosone/s/file?id=wjVg/PLOSOne_formatting_sample_main_body.pdf and

https://journals.plos.org/plosone/s/file?id=ba62/PLOSOne_formatting_sample_title_authors_affiliations.pdf

2. When completing the data availability statement of the submission form, you indicated that you will make your data available on acceptance. We strongly recommend all authors decide on a data sharing plan before acceptance, as the process can be lengthy and hold up publication timelines. Please note that, though access restrictions are acceptable now, your entire data will need to be made freely accessible if your manuscript is accepted for publication. This policy applies to all data except where public deposition would breach compliance with the protocol approved by your research ethics board. If you are unable to adhere to our open data policy, please kindly revise your statement to explain your reasoning and we will seek the editor's input on an exemption. Please be assured that, once you have provided your new statement, the assessment of your exemption will not hold up the peer review process.

If the reviewer comments include a recommendation to cite specific previously published works, please review and evaluate these publications to determine whether they are relevant and should be cited. There is no requirement to cite these works unless the editor has indicated otherwise.

[Note: HTML markup is below. Please do not edit.]

Reviewers' comments:

Reviewer's Responses to Questions

**Comments to the Author**

1. Is the manuscript technically sound, and do the data support the conclusions?

The manuscript must describe a technically sound piece of scientific research with data that supports the conclusions. Experiments must have been conducted rigorously, with appropriate controls, replication, and sample sizes. The conclusions must be drawn appropriately based on the data presented.

Reviewer #1: Yes

Reviewer #2: Partly

Reviewer #3: No

**********

2. Has the statistical analysis been performed appropriately and rigorously?

Reviewer #1: Yes

Reviewer #2: No

Reviewer #3: Yes

**********

3. Have the authors made all data underlying the findings in their manuscript fully available?

The PLOS Data policy requires authors to make all data underlying the findings described in their manuscript fully available without restriction, with rare exception (please refer to the Data Availability Statement in the manuscript PDF file). The data should be provided as part of the manuscript or its supporting information, or deposited to a public repository. For example, in addition to summary statistics, the data points behind means, medians and variance measures should be available. If there are restrictions on publicly sharing data—e.g. participant privacy or use of data from a third party—those must be specified.

Reviewer #1: Yes

Reviewer #2: No

Reviewer #3: Yes

**********

4. Is the manuscript presented in an intelligible fashion and written in standard English?

PLOS ONE does not copyedit accepted manuscripts, so the language in submitted articles must be clear, correct, and unambiguous. Any typographical or grammatical errors should be corrected at revision, so please note any specific errors here.

Reviewer #1: Yes

Reviewer #2: Yes

Reviewer #3: Yes

**********

5. Review Comments to the Author

Please use the space provided to explain your answers to the questions above. You may also include additional comments for the author, including concerns about dual publication, research ethics, or publication ethics. (Please upload your review as an attachment if it exceeds 20,000 characters)

Reviewer #1: The manuscript is written in proper understandable english

Sufficient and consistent Data is presented

Although study is not novel, it's only compilation and presentation of data, but such studies adds information to knowledge panel.

such articles exposes researchers to the Post- COVID complications in respiratory medicine.

Reviewer #2: Study presents surveillance data on 13 respiratory pathogens detected by multiplex real-time PCR from 2,800 ARI cases (ILI and SARI) collected in Quzhou City, Zhejiang Province, China, between November 2023 and July 2024. The authors report an overall positivity rate of 59.28%, with influenza virus, Streptococcus pneumoniae, and adenovirus as the top three pathogens. They describe age-specific, temporal, and geographic distributions and a co-infection rate of 15.68%. The topic is timely and relevant. However, the manuscript has several significant methodological, analytical, and presentation shortcomings that must be addressed before it can be considered for publication.

The following must be addressed as mandatory revisions:

1. Resolve the contradiction in informed consent disclosures and clarify the ethics timeline

2. Correct the data availability statement and deposit data in a public repository

3. Provide sample size justification and describe the enrollment/sampling strategy

4. Address specimen type heterogeneity in the SARI group analytically or acknowledge it explicitly as a major confounder

5. Add Ct value thresholds and quality control details for the PCR assay

6. Provide specific co-infection pair/combination data

7. Perform multivariable analysis or provide explicit justification for why it was not done

8. Revise the title and objectives to accurately reflect the descriptive cross-sectional nature of the study

9. Correct all Chinese-language text in tables and formatting artifacts

10. Add percentage-based detection rates to Table 4

11. Include meaningful figures if possible (temporal trends, age distribution, geographic map)

Reviewer #3: Authors reported the presence of multiple respiratory pathogens post-COVID19 by Real-time PCR. However, the MS did not confirm the presence of these pathogens either by sequencing or by isolation of these pathogens.

**********

6. PLOS authors have the option to publish the peer review history of their article (what does this mean?). If published, this will include your full peer review and any attached files.

If you choose “no”, your identity will remain anonymous but your review may still be made public.

**Do you want your identity to be public for this peer review?** For information about this choice, including consent withdrawal, please see our Privacy Policy.

Reviewer #1: **Yes:** Dr. S Adeeb Mujtaba Ali

Reviewer #2: **Yes:** Ananda Kumar Soshee

Reviewer #3: No

**********

[NOTE: If reviewer comments were submitted as an attachment file, they will be attached to this email and accessible via the submission site. Please log into your account, locate the manuscript record, and check for the action link "View Attachments". If this link does not appear, there are no attachment files.]

To ensure your figures meet our technical requirements, please review our figure guidelines: https://journals.plos.org/plosone/s/figures

You may also use PLOS’s free figure tool, NAAS, to help you prepare publication quality figures: https://journals.plos.org/plosone/s/figures#loc-tools-for-figure-preparation.

NAAS will assess whether your figures meet our technical requirements by comparing each figure against our figure specifications.

Attachment Submitted filename: Comments-PlosOne.pdf

# Author comment: Author response to Decision Letter 1

**DOI:** 10.1371/journal.pone.0346441.r002

16 May 2026

Response to Reviewers

Manuscript ID: PONE-D-26-11954

Title: Shifts in the pathogen spectrum and epidemiology of respiratory tract infections in the post-COVID-19 era: A study from Quzhou, Eastern China (revised title: Pathogen spectrum and epidemiology of respiratory tract infections in Quzhou, Eastern China, from November 2023 to July 2024: a post‑COVID‑19 surveillance study)

Dear Dr. Benjamin M. Liu and the Reviewers,

We sincerely thank the Academic Editor and the reviewers for their careful reading of our manuscript and for their constructive comments. We have revised the manuscript thoroughly in response to all the points raised. Below we provide a point‑by‑point response.

Responses to the Academic Editor’s comments

Comment 1:

The authors stated that "Respiratory specimens used in this study were residual samples collected for routine clinical care purposes between November 2023 and July 2024." This time window covered typical respiratory season in China. Some respiratory viruses, e.g., RSV and flu, have seasonality and prevalence in fall/winter in China. The authors should discuss seasonality and prevalence of these pathogens in fall/winter in northern hemisphere. More references should be cited, with this one (PMID: 40137747) as an example.

Response:

We agree and have expanded the Discussion to address the seasonality of respiratory viruses in the Northern Hemisphere, with a specific focus on influenza and RSV. We now explicitly state that in China, influenza typically peaks from late autumn to early spring (November–March), which aligns with our observations. RSV predominates in autumn and winter in temperate regions. We have cited the recommended reference (Contes & Liu, Pathogens 2025, PMID: 40137747) and added a new reference [22] accordingly.

Location: Discussion, fourth paragraph.

Comment 2:

The authors should introduce or discuss that PCR is just one type (i.e., thermal NAAT) of NAATs for these pathogens and there are isothermal NAATs that are widely used. More references should be cited, with the Liu BM book chapter as an example.

Response:

We have added a brief discussion in the Methods section (subsection 2.3) to clarify that conventional PCR is a thermal‑cycling‑based nucleic acid amplification test (NAAT) and that isothermal NAATs (e.g., LAMP, RPA) are also widely used for rapid detection of respiratory pathogens. The recommended reference (Liu BM, Manual of Molecular Microbiology, ASM Press, 2025) has been cited as reference [10].

Location: Methods, section 2.3, last sentence.

Comment 3:

The authors focused on preventive procedures. Besides, the authors should introduce the impact of viral evolution, e.g., SARS-CoV-2 variants of concern, on the transmission and disease severity. More references should be cited, with this one (PMID: 39744807) as an example.

Response:

We have added a new paragraph in the Discussion (third paragraph) addressing the impact of SARS‑CoV‑2 variants of concern (Alpha, Delta, Omicron) on transmissibility, immune evasion, and disease severity, and how these changes might have indirectly influenced the circulation of other respiratory pathogens. The recommended reference (Liu BM et al., J Med Virol 2025, PMID: 39744807) is now cited as reference [14].

Location: Discussion, third paragraph.

Responses to Reviewer #2

We thank Reviewer #2 for the detailed and constructive critique. All mandatory revisions have been addressed, as detailed below.

Mandatory revision 1 – Resolve the contradiction in informed consent disclosures and clarify the ethics timeline

We have completely rewritten the Ethics Statement (Methods, section 1) to resolve the inconsistency. The study was retrospective and used fully anonymized residual specimens; therefore, the ethics committee waived the requirement for written informed consent. We also explain that ethical approval (24 September 2024) was obtained after the start of data collection (November 2023) but before any analysis was performed (data were accessed for research on 15 January 2025). This is standard for retrospective studies using pre‑existing, de‑identified data.

Location: Methods, section 1 (Ethics statement).

Mandatory revision 2 – Correct the data availability statement and deposit data in a public repository.

We have revised the Data Availability Statement to fully comply with PLOS ONE’s data policy. The anonymized aggregate data supporting the findings of this study are now provided in Supporting Information S1 Table, which has been uploaded as a supplementary file. Patient-level data cannot be publicly shared due to patient privacy protection. De-identified individual data may be available from the corresponding author upon reasonable request, subject to approval by the institutional ethics committee. This statement has been included at the end of the manuscript (Data Availability Statement section).

Location: Data Availability Statement section.

Mandatory revision 3 – Provide sample size justification and describe the enrollment/sampling strategy

We have added a detailed description in Methods 2.1. All consecutive ARI patients meeting the case definitions at the participating county‑level hospitals during the study period were included; no additional sampling or selection was applied. The ILI:SARI ratio (1,960:840) reflects the natural distribution in our surveillance network. A post‑hoc power analysis shows that with 2,800 cases, the study had 84% power to detect a 10% absolute difference in positivity rates between subgroups (α=0.05, two‑sided).

Location: Methods, section 2.1, paragraphs 2–3.

Mandatory revision 4 – Address specimen type heterogeneity in the SARI group analytically or acknowledge it explicitly as a major confounder

We have added a new subsection 2.5 in Methods to describe the specimen types:among SARI cases, the majority (83.6%, 702/840) were throat swabs; bronchoalveolar lavage fluid (BALF, n=130) and pleural fluid (n=8) were used in a minority. Given the small number of non throat swab specimens (n=138, 16.4%), formal statistical comparison was not performed.

Location: Methods, section 2.5; and Limitations (Discussion, third point).

Mandatory revision 5 – Add Ct value thresholds and quality control details for the PCR assay

We have added a new subsection 2.4 in Methods that describes: a sample as positive when Ct <35; Ct values 35–38 were re‑tested and considered borderline positive if still <38; inclusion of positive/negative extraction controls, no‑template control, and no‑reverse‑transcriptase control for RNA pathogens; assessment of inter‑run variability (CV <5% for all targets).

Location: Methods, section 2.4.

Mandatory revision 6 – Provide specific co‑infection pair/combination data

We have added detailed co‑infection data in the Results (section 2.2, within the parentheses). For dual infections, the most frequent combinations were RV+SP, AdV+SP, and RSV+SP. For triple infections, SARS‑CoV‑2+RV+SP and SP+RV+AdV predominated. Quadruple and higher‑order co‑infections were also observed, with examples provided.

Location: Results, section 2.2, after Table 2.

Mandatory revision 7 – Perform multivariable analysis or provide explicit justification for why it was not done

We have performed a multivariable binary logistic regression analysis. The dependent variable was overall positivity; independent variables included age group, sex, case type, month, and region. Adjusted odds ratios with 95% CIs are reported. Age ≤5 years, ILI cases, and certain regions (Kaihua, Kecheng, Longyou, Qujiang) were independent predictors of higher positivity. The Hosmer‑Lemeshow test indicated good model fit (χ²=8.24, P=0.41). This is now presented in Results section 5 and described in Methods section 4.

Location: Methods, section 4 (Statistical Analysis); Results, section 5 (Multivariable analysis).

Mandatory revision 8 – Revise the title and objectives to accurately reflect the descriptive cross‑sectional nature of the study

We have revised the title to: "Pathogen spectrum and epidemiology of respiratory tract infections in Quzhou, Eastern China, from November 2023 to July 2024: a post‑COVID‑19 surveillance study" The objectives in the Abstract and Introduction have been rephrased to reflect a descriptive surveillance study rather than claiming to demonstrate a "shift" without local pre‑pandemic data.

Location: Title page; Abstract, Objective; Introduction, last paragraph.

Mandatory revision 9 – Correct all Chinese‑language text in tables and formatting artifacts

All Chinese characters have been removed from tables. Sex categories are now "Male/Female". Abbreviations are consistent (e.g., AdV, Flu, MP, SP). Table formatting has been standardized.

Location: Tables 1–5.

Mandatory revision 10 – Add percentage‑based detection rates to Table 4

Table 4 now presents both counts and percentages for each pathogen per month. For example, "46.18%" is shown alongside the absolute number.

Location: Table 4.

Mandatory revision 11 – Include meaningful figures (temporal trends, age distribution, geographic map)

We have added two figures: Figure 1 (positivity rates of common pathogens in different age groups) and Figure 2 (monthly positivity trends of major pathogens from November 2023 to July 2024). Due to the regulatory requirement in China that administrative boundary maps at the county/district level must undergo official review and approval prior to publication, a geographic map is not included in this manuscript. Moreover, given that our geographic analysis involves only six discrete counties/districts with clear numerical differences, a choropleth map would not add substantial information beyond the detailed numerical comparisons already presented in Results section 3.3. Therefore, we report the geographic distribution data in tabular form with explicit positivity rates for each region.

Location: Results, sections 3.1 and 3.2; Figures 1 and 2.

Additional major concerns raised by Reviewer #2 – we have also addressed the following:

· 1.2 Marked temporal imbalance (July n=30): We have now explicitly added a sentence in the Limitations section acknowledging that the small sample size in July (n=30) limits the reliability of the positivity rate estimate for that month.

Location: Discussion, Limitations (second point).

· 1.3 ILI/SARI definitions: We have revised the SARI definition to include clinical evidence of lower respiratory tract involvement (shortness of breath, tachypnea, abnormal breath sounds) following WHO and Zhejiang Provincial Protocol.

Location: Methods, section 2.1.

· 1.4 Missing summer‑autumn season: We clearly state in the Limitations that the study period covered only nine months and therefore seasonal descriptions are preliminary and require full annual data.

Location: Discussion, Limitations (first point).

· 2.3 SP detection – colonization vs. infection: We added a cautionary note in Results (section 2.2) and in the Limitations that SP detection by nasopharyngeal PCR cannot distinguish colonization from infection and may overestimate the true infectious burden.

Location: Results, section 2.2 (parentheses after SP data); Discussion, Limitations.

· 3.3 Multiple comparisons: We have added a sentence in the Statistical Analysis section that, given the exploratory nature of this study, no correction for multiple comparisons was applied; findings with borderline P values should be interpreted cautiously.

Location: Methods, section 4, last sentence.

· 3.4 Trend test for co‑infection vs. age: The Cochran‑Armitage trend test result (χ²=41.2, P<0.001) is now explicitly reported in Results section 3.1, showing that the co‑infection rate decreased significantly with age.

Location: Results, section 3.1, last sentence before Table 3.

· 5.2 Geographic variation – unsupported speculation: We have removed the speculative statement about "differences in professional expertise". We now simply report the observed differences and note that multiple factors (population density, age structure, healthcare‑seeking behavior, true epidemiological differences) may contribute.

Location: Results, section 3.3; Discussion has no such speculation.

· 6.4 Discussion depth: We have substantially expanded the Discussion to include international literature on post‑pandemic respiratory pathogen dynamics, immunity debt, viral evolution, and the seasonality of respiratory viruses. Additional references have been added.

Location: Discussion, entire section.

· 6.6 Lack of clinical outcome data: We have explicitly stated in Methods (2.1) that clinical outcome data (hospitalization duration, ICU admission, mortality) were not collected, and we have listed this as a limitation in the Discussion.

Location: Methods, section 2.1, last sentence; Discussion, Limitations (eighth point).

Responses to Reviewer #3

Comment:

Authors reported the presence of multiple respiratory pathogens post‑COVID‑19 by Real‑time PCR. However, the MS did not confirm the presence of these pathogens either by sequencing or by isolation of these pathogens.

Response:

We thank the reviewer for this important point. We have added a specific limitation in the Discussion (ninth point) acknowledging that we did not further confirm positive PCR results by sequencing or culture. PCR detects nucleic acids and does not prove the presence of viable pathogens or distinguish colonization from infection for certain bacteria (e.g., S. pneumoniae). Future studies should incorporate culture or sequencing for stronger etiological confirmation.

Location: Discussion, Limitations (ninth point).

Responses to Reviewer #1

Reviewer #1 provided positive feedback with no specific revision requests. We thank the reviewer for the encouraging comments and for recognizing the value of our surveillance data.

Additional revisions made to comply with journal requirements

1. PLOS ONE style requirements: The manuscript has been formatted according to the PLOS ONE templates. All file naming will follow journal guidelines upon submission.

2. Data availability statement: Revised as described above.

3. Ethics approval timeline: Fully clarified in the Ethics Statement.

4. ORCID iD: The corresponding authors have verified their ORCID iDs.

5.Data availability and supplementary file: We have compiled all anonymized aggregate data into Supporting Information S1 Table and submitted it. The Data Availability Statement has been updated to indicate that the aggregate data are fully available in this supplementary file, while patient level data remain restricted to protect patient privacy.

Summary of changes

· Title modified to reflect descriptive surveillance.

· Methods expanded with specimen type breakdown, Ct thresholds, quality control, sample size justification, and multivariable analysis.

· Results updated with co‑infection pair data, trend test results, and multivariable regression.

· Tables 1–5 cleaned of Chinese characters and formatting errors; Table 4 now includes percentages.

· Figures 1 and 2 added.

· Discussion substantially rewritten to address seasonality, viral evolution, immunity debt, and limitations including small July sample, lack of pre‑pandemic baseline, and absence of sequencing/isolation confirmation.

· Ethical and data availability statements corrected.

We believe the manuscript has been substantially improved and now meets PLOS ONE’s publication criteria. We thank the editor and reviewers again for their time and valuable guidance.

We look forward to your favorable decision.

Sincerely,

Rui-jun Yang, Min Wang, Shi-teng Huang, Bing-dong Zhan (on behalf of all authors)

Attachment Submitted filename: Response to Reviews(20260515).docx

# Editor report: Decision Letter 1

**DOI:** 10.1371/journal.pone.0346441.r003

**Authors:** Benjamin Liu

20 May 2026

Pathogen spectrum and epidemiology of respiratory tract infections in Quzhou, Eastern China, from November 2023 to July 2024: a post‑COVID‑19 surveillance study

PONE-D-26-11954R1

Dear Dr. Yang,

We’re pleased to inform you that your manuscript has been judged scientifically suitable for publication and will be formally accepted for publication once it meets all outstanding technical requirements.

Within one week, you’ll receive an e-mail detailing the required amendments. When these have been addressed, you’ll receive a formal acceptance letter and your manuscript will be scheduled for publication.

An invoice will be generated when your article is formally accepted. Please note, if your institution has a publishing partnership with PLOS and your article meets the relevant criteria, all or part of your publication costs will be covered. Please make sure your user information is up-to-date by logging into Editorial Manager at Editorial Manager® and clicking the ‘Update My Information' link at the top of the page. For questions related to billing, please contact billing support.

If your institution or institutions have a press office, please notify them about your upcoming paper to help maximize its impact. If they’ll be preparing press materials, please inform our press team as soon as possible -- no later than 48 hours after receiving the formal acceptance. Your manuscript will remain under strict press embargo until 2 pm Eastern Time on the date of publication. For more information, please contact onepress@plos.org.

Kind regards,

Benjamin M. Liu, PhD, D(ABMM), MB(ASCP)

Academic Editor

PLOS One

Additional Editor Comments (optional):

Reviewers' comments:

# Editor report: Acceptance letter

**DOI:** 10.1371/journal.pone.0346441.r004

**Authors:** Benjamin Liu

PONE-D-26-11954R1

PLOS One

Dear Dr. Yang,

I'm pleased to inform you that your manuscript has been deemed suitable for publication in PLOS One. Congratulations! Your manuscript is now being handed over to our production team.

At this stage, our production department will prepare your paper for publication. This includes ensuring the following:

* All references, tables, and figures are properly cited

* All relevant supporting information is included in the manuscript submission,

* There are no issues that prevent the paper from being properly typeset

You will receive further instructions from the production team, including instructions on how to review your proof when it is ready. Please keep in mind that we are working through a large volume of accepted articles, so please give us a few days to review your paper and let you know the next and final steps.

Lastly, if your institution or institutions have a press office, please let them know about your upcoming paper now to help maximize its impact. If they'll be preparing press materials, please inform our press team within the next 48 hours. Your manuscript will remain under strict press embargo until 2 pm Eastern Time on the date of publication. For more information, please contact onepress@plos.org.

You will receive an invoice from PLOS for your publication fee after your manuscript has reached the completed accept phase. If you receive an email requesting payment before acceptance or for any other service, this may be a phishing scheme. Learn how to identify phishing emails and protect your accounts at https://explore.plos.org/phishing.

If we can help with anything else, please email us at customercare@plos.org.

Thank you for submitting your work to PLOS ONE and supporting open access.

Kind regards,

PLOS ONE Editorial Office Staff

on behalf of

Dr. Benjamin M. Liu

Academic Editor

PLOS One
